# Global and Chinese growth failure disease burden analysis and projections for adolescents and children, 1990–2021

**DOI:** 10.3389/fpubh.2025.1639801

**Published:** 2025-10-09

**Authors:** Zhifei Wu, Runbing Xu, DongKai Qiu, Haiyan Wang, Jiajing Zheng

**Affiliations:** ^1^Department of Pediatrics, Beilun Branch of the First Affiliated Hospital, College of Medicine, Zhejiang University, Ningbo, China; ^2^Department of Pediatrics, Beilun People's Hospital, Ningbo, China; ^3^Beijing University of Chinese Medicine, Beijing, China

**Keywords:** growth failure, stunting, wasting, underweight, burden of disease

## Abstract

**Objective:**

This study aimed to comprehensively analyze the global and Chinese burden of growth failure (GF) in children and adolescents from 1990 to 2021, focusing on mortality, disability-adjusted life years (DALYs), years lived with disability (YLDs), and years of life lost (YLLs). The study also explored disparities across gender, age, socio-demographic index (SDI) regions, and geographic locations to inform targeted interventions.

**Design:**

A retrospective analysis was conducted using data from the Global Burden of Disease (GBD) 2021 database. Trends in deaths, DALYs, YLDs, YLLs, and their age-standardized rates (ASRs) were evaluated. Decomposition, health inequality, and prediction analyses were performed to assess contributing factors and future trends.

**Participants:**

Children and adolescents aged 0–19 years, with emphasis on those under 5 years, were included in the analysis.

**Results:**

From 1990 to 2021, global deaths, DALYs, YLDs, and YLLs for GF declined by 78.17, 77.86, 49.71, and 78.20%, respectively. China exhibited even more pronounced declines (98.16, 98.15, 97.10, and 98.15%). The burden was concentrated in children under 5, with males disproportionately affected. Low-SDI regions, particularly Western Sub-Saharan Africa and South Asia, accounted for over 60% of the global burden, with ASRs up to 1,000 times higher than in high-SDI regions.

**Conclusion:**

Despite substantial progress, GF remains a critical public health challenge, with pronounced disparities persisting in low-SDI regions. Urgently needed are targeted interventions—particularly for children under 5 in these settings—to address the inequitable burden. These conclusions should be interpreted in light of key limitations: the analysis relies on modeled GBD 2021 estimates that may be biased due to data sparsity and cause-redistribution assumptions in low-SDI contexts; furthermore, incidence/prevalence and intervention coverage were not assessed.

## Introduction

Childhood growth failure (HF) remains a significant public health issue in developing countries. It is estimated that nearly half of all deaths in children under 5 years of age globally can be attributed to HF, with stunting, wasting, and underweight being the three most common indicators of this condition (1–3). Recent statistics show that 149 million children under five are stunted, 49.5 million are wasted, and an additional 15.9 million exhibit both forms of HF (4–6). The prevalence of these conditions varies by region, with growth retardation being the most widespread, followed by underweight and wasting (1, 7, 8). In China, the burden of growth failure among children and adolescents has declined markedly since 1990, yet important challenges remain. In 2021, China recorded 5,164 deaths (95% UI: 3,420–7,696), 463,844 DALYs (95% UI, 304,639–692,635), 4,242 YLDs (95% UI, 182–9,935), and 459,602 YLLs (95% UI, 304,254–685,053), corresponding to age-standardized rates of 1.8, 161.53, 1.43, and 160.1 per 100,000, respectively. Compared with 1990, these represent declines of 98.16, 98.15, 97.10, and 98.15%. Growing evidence indicates that wasting and growth retardation often coexist within the same child and are interrelated, driven by common factors, which further heighten the risk of mortality (6). Previous studies estimated that wasting and growth retardation together account for 14.8 and 12.6% of the disability-adjusted life years (DALYs) in children under 5 years of age globally (9). A recent large-scale cross-sectional survey revealed that all children with both wasting and growth retardation were also underweight (10). Numerous studies have demonstrated that HF not only increases the risk of infectious diseases, cognitive developmental delays, and chronic non-communicable diseases (e.g., diabetes and cardiovascular diseases), but it also reduces labor productivity in adulthood. Moreover, it is strongly associated with lower levels of education and employment, thereby exacerbating the intergenerational transmission of poverty (2, 11, 12). Additionally, children with growth retardation may experience defects in organ size, which can negatively impact physiological functioning (13).

In recent years, several epidemiological studies have assessed the prevalence, contributing factors, and long-term health consequences of childhood HF, particularly growth retardation, at global, regional, and national levels (1, 14–17). However, existing studies have several limitations. Most focus only on specific countries or regions and lack dynamic assessments on a global scale. Additionally, there is insufficient analysis of long-term trends across different age groups of children and adolescents. The Global Burden of Disease (GBD) 2021 dataset, which covers the incidence, morbidity, mortality, and DALYs of 371 diseases and injuries across 204 countries, as well as 88 behavioral, environmental, occupational, and metabolic risk factors (18), provides a comprehensive framework for a more in-depth analysis. The strengths of the GBD dataset lie in its use of harmonized diagnostic criteria and methods, ensuring high-quality and comparable data, and its ability to systematically track the global and regional burden of disease over time. Building on this dataset, this study aims to conduct a thorough analysis of global adolescent and childhood HF. However, prior studies typically concentrate on a single indicator (e.g., stunting prevalence) or a narrow set of geographies, and rarely present long-term, age- and sex-disaggregated trends across multiple burden metrics (deaths, DALYs, YLDs, YLLs) accompanied by an explicit decomposition of underlying drivers. The study will assess differences in burden and trends across gender, age groups, regions, and socio-economic backgrounds, explore possible influencing factors, and predict future trends. Comparisons will also be made with data from China.

## Methods

### Data acquisition and sources

This study used the Global Burden of Disease (GBD) 2021 data to assess the global impact of growth failure for adolescents and children. The GBD 2021 dataset is accessible through the Global Health Data Exchange (GHDx) Results Tool.^1^ It provides open access to detailed global and regional health metrics. The dataset estimates 371 diseases and injuries, covering incidence, prevalence, mortality, and Disability-Adjusted Life Years (DALYs). Additionally, it includes 88 risk factors pertaining to behavioral, environmental, occupational, and metabolic domains. This analysis focused on the mortality and DALYs associated with growth failure for adolescents and children in the worldwide. DALYs, which combine years lived with disability (YLDs) and years of life lost (YLLs) due to early death, serve as a measure for evaluating both fatal and nonfatal disease impacts. The Socio-population Index (SDI) is a composite measure of socioeconomic development that combines total fertility rates, mean years of education, and lagged per capita income for individuals aged 20 and younger across various regions. The SDI ranges from 0 to 1, classifying countries into five tiers: low, low-middle, middle, high-middle, and high. The study analyzed data from 204 countries and territories from 1990 to 2021. It provided a comprehensive trend analysis and an in-depth evaluation of the global burden of growth failure for adolescents and children. Ethical approval and informed consent were not necessary since this study utilized publicly available data. The research adhered to guidelines for accurate and transparent reporting in health assessments.

#### Study population, inclusion and exclusion criteria

Population and period: We included all GBD 2021 estimates for children and adolescents aged 0–19 years across 204 countries and territories, 21 GBD regions, and 5 SDI quintiles for the years 1990–2021. Outcomes and metrics: We included location-year-age-sex estimates with available point estimates and 95% uncertainty intervals for deaths, DALYs, YLDs, and YLLs attributable to growth failure and its components. Estimates with missing values or without uncertainty intervals were excluded from analysis and figure/tabulation (1). Age/sex stratification: We analyzed both sexes combined and male/female separately, using standard GBD 5-year age bands (<5, 5–9, 10–14, 15–19 years). Records outside 0–19 years were excluded. Location coverage: All locations with complete GBD 2021 estimates for the specified outcomes and age range were retained. Subnational locations were included when available and aggregated consistently to national and regional levels; locations without complete time series for 1990–2021 were excluded from trend analyses but retained for cross-sectional 2021 summaries if complete for that year. Internal consistency: For each location-age-sex-year, we required internal consistency between component metrics (e.g., DALYs = YLLs + YLDs within GBD rounding/uncertainty). Records failing basic consistency checks were excluded from sensitivity analyses. Data provenance and modeling: We used the finalized GBD 2021 results produced through standardized cause-of-death ensemble modeling, DisMod-MR 2.1 for non-fatal outcomes, and codcorrect/cause-redistribution procedures. We did not introduce additional external data or re-estimate parameters; thus, inclusion/exclusion pertains to the analytical dataset extracted from the GBD 2021 Results Tool. A concise flow description has been added to the Methods to show how GBD outputs were filtered to the final analytic dataset (18).

#### Operational definitions of key variables

We followed GBD 2021 and WHO child growth standards for definitional criteria: Growth failure (GF): We use GF as an umbrella term encompassing three anthropometric deficits in children: stunting, wasting, and underweight, as defined below. In burden estimates, GF reflects the aggregate burden from these nutrition-related growth deficits among children and adolescents, quantified via deaths, YLLs, YLDs, and DALYs in GBD 2021. Stunting: Height-for-age z-score (HAZ) < −2 standard deviations relative to the WHO Child Growth Standards median for age and sex (0–59 months). For older children/adolescents, GBD harmonizes survey-based anthropometry using WHO reference standards and DisMod-MR 2.1 to produce comparable non-fatal estimates. Wasting: Weight-for-height z-score (WHZ) < −2 SD relative to the WHO Child Growth Standards median for age and sex (0–59 months). For children measured by MUAC in surveys, GBD crosswalks MUAC to WHZ using established conversion models. Severe wasting corresponds to WHZ < −3 SD. Underweight: Weight-for-age z-score (WAZ) < −2 SD relative to the WHO Child Growth Standards median for age and sex (0–59 months). Severe underweight corresponds to WAZ < −3 SD (19, 20).

YLLs: Years of Life Lost due to premature mortality, computed as the product of deaths and standard life expectancy at age of death per GBD reference life table. YLDs: Years Lived with Disability, computed as prevalence of sequelae multiplied by disability weights derived from GBD population surveys. DALYs: Disability-Adjusted Life Years, calculated as DALYs = YLLs + YLDs. Age-standardized rates (ASRs): Direct age-standardization to the GBD world standard population, expressed per 100,000 population. SDI: Composite index of lag-distributed income per capita, average educational attainment in those aged 15+, and total fertility rate under age 25, scaled 0–1 and grouped into five quintiles (low to high).

Data sources: Nationally representative household surveys (e.g., DHS, MICS), nutrition surveillance, and vital registration systems compiled by GBD. Anthropometric indicators were standardized to WHO definitions; non-fatal estimates used DisMod-MR 2.1 to ensure internal consistency across incidence, prevalence, remission, and mortality. Cause of death and redistribution: GBD applied standardized garbage-code redistribution and codcorrect to align cause-specific deaths with all-cause mortality envelopes. Uncertainty: 95% uncertainty intervals derive from 1,000 posterior draws propagated through all computation steps.

### Population analysis and global burden analysis

We analyzed age-standardized Deaths, DALYs, YLDs, YLLs and their 95% uncertainty intervals for growth failure for adolescents and children using data from the GBD 2021 study, which included 204 countries, 21 GBD regions, and 5 Socio-Demographic Index (SDI) quintiles. Additionally, we stratified the Deaths, DALYs, YLDs, YLLs by sex into two groups. We then divided the population into 4 age groups (<5 years, 5–9 years…) based on a 5-year cycle. The study utilized high-resolution maps to visualize the global burden of HF disease, emphasizing disparities across socio-demographic and geographic contexts.

### Decomposition analysis

We used decomposition analysis to measure how population growth, aging, and epidemiological changes contributed to trends in total Deaths, DALYs, YLDs, YLLs. This approach provided clearer insights into how demographic and health system factors influence the disease burden. This study uses a method consistent with the analytical framework of previous Global Burden of Disease (GBD) studies. These studies estimate how changes in population structure and risk factors affect shifts in disease outcomes.

### Health inequality analysis

Health inequality analysis examines disparities in disease burden across countries and regions, helping to shape public health policies. This analysis employs the slope index of inequality (SII) and the concentration index (CII) to measure health inequalities. The SII illustrates the relationship between health indicators and socioeconomic status. It utilizes the Sociodemographic Index (SDI) in a linear regression analysis. The CII ranges from −1 to 1 and indicates the variation of health outcomes based on economic status. Values closer to 0 reflect less inequality, positive values favor the wealthy, and negative values favor the impoverished. This study calculated the SII and CII for the Mortality and DALYs of caries in anxiety, depression, bipolar disorder, and schizophrenia between 1990 and 2021. It emphasizes health inequalities in a global context, across various regions, and among 204 countries.

### Prediction analysis

To better formulate public health policies and allocate medical resources, we divided the population into gender-based subgroups (females and males) and used the Bayesian-Aperiodic-People-Cohort (BAPC) model to predict trends in the incidence and prevalence of growth failure for adolescents and children over the next 15 years. By considering temporal variations and age-specific trends, these models provide a reliable and comprehensive outlook on the future burden of growth failure. Studies have shown that combining the Integrated Nested Laplace Approximation (INLA) with the BAPC model effectively approximates marginal posterior distributions, thereby avoiding the mixing and convergence issues typically associated with the Markov Chain Monte Carlo (MCMC) sampling technique used in traditional methods.

### Statistical software

All statistical analyses were conducted using R (version 4.3.3) and Stata 18 (StataCorp, College Station, TX, USA). We created custom scripts to perform decomposition and sensitivity analyses. We performed Bayesian analysis using WinBUGS (version 1.4) as part of our analytical process. Geographic and spatial analyses were conducted with ArcGIS Pro and QGIS (version 3.16), allowing for the creation of high-resolution maps that visualize the TBL burden and disparities. We generated data visualizations, including bi-lateral and two-axis plots, using the ‘ggplot2’ and ‘Benchmarking’ packages in R.

### Statistical significance

In this study, we set the *p* < 0.05 threshold for all analyses to determine statistical significance. This approach aligns with standard practices in epidemiological and public health research, especially concerning studies on the Global Burden of Disease.

## Results

### Comparison of the current status of the disease burden of growth failure in children and adolescents globally and in China and the trend of change from 1990 to 2021

**Figure 1:**
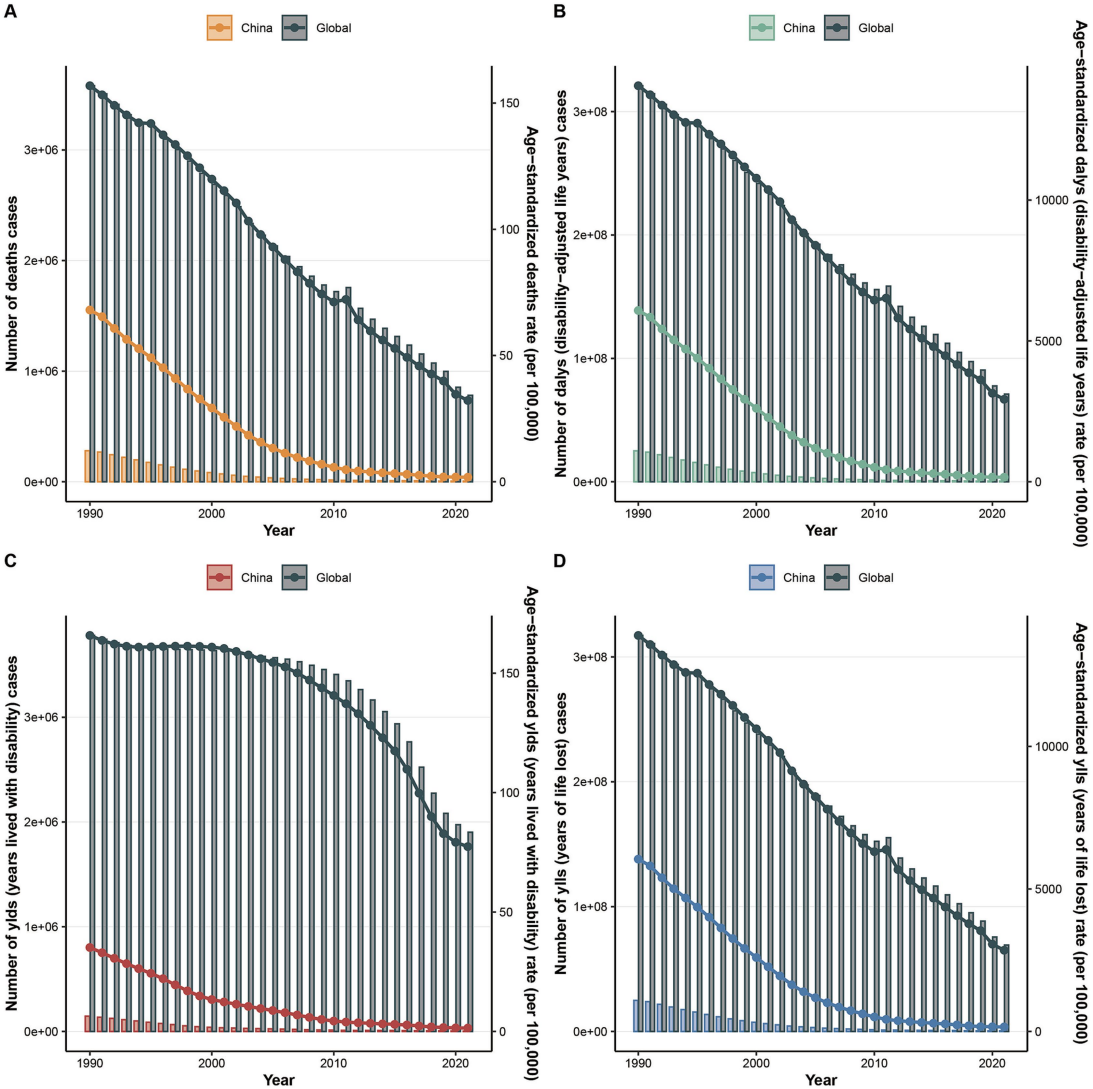
The changes in the number of indicators and their Age-Standardized Rates (ASRs) for the global and Chinese burden of growth failure-related diseases among children and adolescents from 1900 to 2021 (**A.** Deaths; **B.** DALYs; **C.** YLDs; **D.** YLLs).

From 1990 to 2021, the number of deaths, DALYs, YLDs, and YLLs, along with their corresponding ASRs, for adolescent and pediatric HF showed a consistent decline, both globally and in China. Notably, ASRs in China were significantly lower than the global average, with the most pronounced decline occurring before 2005—much earlier than the global trend (Figure 1). Globally, all growth failure burden indicators showed substantial declines from 1990 to 2021, with deaths and YLLs declining by approximately 78% and DALYs by 78%. China demonstrated even more dramatic improvements, with reductions exceeding 98% across all metrics. China’s burden represented less than 1% of global DALYs and YLLs, with age-standardized rates substantially below global averages, reflecting significant progress in addressing childhood growth failure.

### Global and China child and adolescent growth failure disease burden and trends across different subgroups (sex, age, SDI, region, and country)

**Figure 2:**
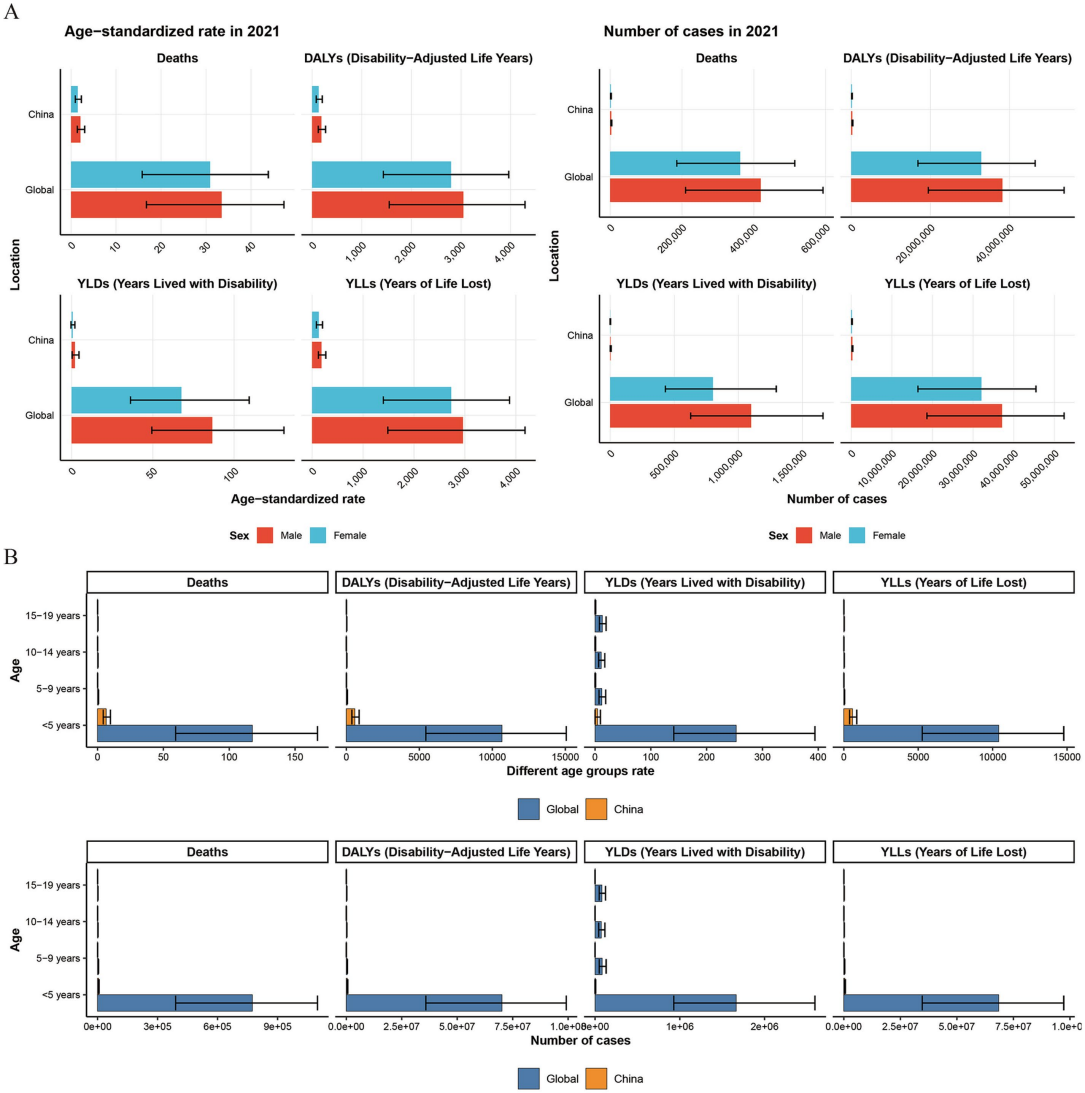
The comparison of the number of indicators and their Age-Standardized Rates (ASRs) for the global and Chinese burden of growth failure-related diseases among children and adolescents of different genders and age groups in 1990 and 2021 (**A.** Different genders; **B.** Different age groups).

Between 1990 and 2021, child and adolescent HF disease burden and trends exhibited notable regional and national variations. In 2021, both globally and in China, males consistently exhibited higher mortality rates, DALYs, YLDs, and YLLs compared to females. This disparity was particularly pronounced in YLDs, where the male-to-female ratio was 1.4:1 globally and 3.5:1 in China for mortality, and 1.3:1 globally and 2.9:1 in China for ASRs (Figure 2A).

Regarding age groups, HF-related deaths, DALYs, YLDs, and YLLs were predominantly concentrated in children under 5 years of age globally. The respective proportions of these outcomes in this age group were 98.98, 98.78, 87.52, and 99.09%. The age-standardized rates for these outcomes were over three times higher than the global average and approximately 200 times higher than in other age groups (excluding YLDs, where the discrepancy was around 20 times). A similar pattern was observed in China prior to 2010. However, in the last decade, with improvements in living and healthcare conditions, the age-related disparity has diminished (Figure 2B, Supplementary Figure S1, and Tables 1–4).

**Table 1:** Burden of deaths due to growth failure in children and adolescents (1990 and 2021).

Location	Rate per 100,000 (95%UI)				
	1990		2021		1990–2021
	Deaths cases	The age-standardized deaths rate	Deaths cases	The age-standardized deaths rate	EAPC
Global	3,580,631 (2,574,094–4,189,794)	156.85 (112.76–183.54)	781,528 (397,299–1,108,644)	32.21 (16.36–45.7)	−4.9 (−5.2 to −4.6)
SDI region					
High SDI	6,549 (4,592–8,523)	2.88 (2.02–3.75)	624 (371–924)	0.31 (0.18–0.46)	−6.03 (−6.34 to −5.71)
High-middle SDI	102,501 (75,619–127,529)	29.94 (22.09–37.26)	4,032 (2,664–5,477)	1.55 (1.03–2.11)	−9.4 (−9.63 to −9.17)
Middle SDI	652,274 (478,257–774,448)	88.3 (64.74–104.84)	60,887 (37,498–83,008)	9.33 (5.74–12.73)	−6.62 (−6.8 to −6.43)
Low-middle SDI	1,407,916 (1,081,317–1,629,239)	220.48 (169.33–255.15)	205,574 (122,880–280,325)	29.11 (17.4–39.7)	−6.06 (−6.37 to −5.76)
Low SDI	1,409,408 (956,870–1,674,451)	422.09 (286.62–501.52)	509,830 (234,467–736,295)	83.65 (38.48–120.8)	−5.08 (−5.34 to −4.81)
GBD region					
Andean Latin America	21,892 (16,420–26,634)	112.59 (84.45–136.98)	1,901 (1,240–2,737)	8.37 (5.46–12.05)	−7.73 (−7.93 to −7.53)
Australasia	39 (25–61)	0.68 (0.43–1.08)	7 (4–11)	0.1 (0.06–0.17)	−3.9 (−4.51 to −3.3)
Caribbean	17,283 (12,186–21,424)	113.63 (80.12–140.86)	5,083 (3,147–7,065)	35.65 (22.06–49.55)	−3.27 (−3.59 to −2.95)
Central Asia	38,007 (27,768–45,079)	108.36 (79.17–128.52)	5,624 (3,846–7,659)	15.28 (10.45–20.81)	−6.12 (−6.5 to −5.73)
Central Europe	5,313 (3,823–6,587)	15.8 (11.36–19.58)	318 (195–446)	1.54 (0.95–2.16)	−6.75 (−7.05 to −6.46)
Central Latin America	61,571 (45,141–73,023)	72.69 (53.3–86.2)	6,096 (4,063–8,516)	8.16 (5.42–11.42)	−6.26 (−6.47 to −6.05)
Central Sub-Saharan Africa	147,950 (74,639–196,956)	387.49 (195.6–515.9)	41,881 (6,923–76,947)	54.03 (8.95–99.24)	−6.25 (−6.87 to −5.62)
East Asia	283,153 (206,849–346,897)	66.4 (48.51–81.35)	5,401 (3,588–8,029)	1.82 (1.21–2.71)	−12.77 (−13.63 to −11.9)
Eastern Europe	5,715 (4,236–6,973)	9 (6.67–10.98)	405 (278–543)	1.08 (0.74–1.45)	−6.9 (−7.41 to −6.38)
Eastern Sub-Saharan Africa	553,622 (377,382–666,081)	417.7 (284.83–502.64)	149,807 (78,674–219,596)	63.82 (33.53–93.53)	−6.04 (−6.36 to −5.72)
High-income Asia Pacific	677 (459–896)	1.79 (1.21–2.37)	59 (36–84)	0.24 (0.15–0.35)	−5.07 (−5.43 to −4.71)
High-income North America	722 (448–1,073)	0.9 (0.56–1.34)	207 (131–316)	0.27 (0.17–0.41)	−2.99 (−3.25 to −2.73)
North Africa and Middle East	192,693 (143,803–243,867)	102.16 (76.24–129.3)	24,680 (16,817–32,034)	10.95 (7.46–14.21)	−6.91 (−7.19 to −6.64)
Oceania	5,682 (4,214–7,264)	153.68 (113.96–196.46)	4,615 (3,073–6,268)	64.8 (43.15–88.02)	−1.95 (−2.25 to −1.66)
South Asia	1,219,248 (987,375–1,405,425)	210.89 (170.78–243.1)	150,131 (108,038–194,820)	25.68 (18.48–33.33)	−6.03 (−6.38 to −5.67)
Southeast Asia	295,782 (225,379–349,325)	137.75 (104.96–162.69)	28,410 (19,002–36,825)	13.68 (9.14–17.73)	−6.88 (−7 to −6.76)
Southern Latin America	2,566 (1,926–3,183)	13.53 (10.15–16.79)	191 (128–274)	1.19 (0.79–1.71)	−6.72 (−7.13 to −6.31)
Southern Sub-Saharan Africa	39,888 (28,791–47,341)	144.99 (104.65–172.08)	14,825 (10,017–19,769)	50.06 (33.82–66.76)	−2.67 (−3.12 to −2.23)
Tropical Latin America	47,447 (31,339–58,776)	75.37 (49.77–93.38)	1,903 (1,240–2,657)	2.99 (1.95–4.18)	−9.53 (−9.81 to −9.25)
Western Europe	650 (383–994)	0.77 (0.45–1.17)	111 (55–181)	0.14 (0.07–0.23)	−4.19 (−4.51 to −3.87)
Western Sub-Saharan Africa	640,730 (396,381–789,657)	487.03 (301.33–600.25)	339,874 (131,653–504,460)	115.47 (44.75–171.37)	−4.49 (−4.78 to −4.19)

EAPC, estimated annual percentage change; SDl, Sociodemographic Index; Ul, uncertainty interval. “EAPC is expressed as 95% CIs.

**Table 2:** Burden of DALYs due to growth failure in children and adolescents (1990 and 2021).

Location	Rate per 100,000 (95%UI)				
	1990		2021		1990–2021
	DALYs cases	The age-standardized DALYs rate	DALYs cases	The age-standardized DALYs rate	EAPC
Global	320,961,861 (229,949,302–375,935,595)	14,059.83 (10,073.14–16,468.1)	71,049,289 (36,584,453–100,207,771)	2,927.51 (1,506.27–4,129.64)	−4.85 (−5.16 to −4.55)
SDI region					
High SDI	615,979 (425,893–810,318)	270.33 (186.94–355.29)	82,789 (44,372–131,318)	39.39 (21.06–62.31)	−5.04 (−5.36 to −4.72)
High-middle SDI	9,246,064 (6,787,006–11,501,112)	2,700.86 (1,982.37–3,359.69)	397,697 (263,370–543,198)	152.89 (101.13–208.85)	−9.14 (−9.36 to −8.92)
Middle SDI	58,838,689 (42,975,266–69,697,281)	7,965.17 (5,817.47–9,435.15)	5,764,626 (3,606,997–7,797,428)	882.42 (551.42–1,193.98)	−6.48 (−6.66 to −6.29)
Low-middle SDI	126,540,805 (97,243,327–146,238,113)	19,817.23 (15,228.83–22,903.32)	18,970,429 (11,429,673–25,649,639)	2,685.42 (1,617.33–3,631.2)	−5.98 (−6.28 to −5.68)
Low SDI	125,542,771 (85,043,371–149,306,509)	37,595.83 (25,473.23–44,716.59)	45,781,212 (21,206,233–66,073,378)	7,511.4 (3,480.71–10,839.85)	−5.05 (−5.32 to −4.79)
GBD region					
Andean Latin America	1,951,365 (1,448,797–2,378,224)	10,035.67 (7,451.28–12,230.98)	169,483 (109,546–244,332)	746.09 (482.09–1,075.78)	−7.73 (−7.93 to −7.53)
Australasia	3,501 (2,146–5,605)	61.57 (37.72–98.6)	616 (325–1,209)	9.13 (4.83–17.67)	−3.83 (−4.42 to −3.23)
Caribbean	1,541,112 (1,083,763–1,907,913)	10,132.16 (7,125.62–12,543.81)	454,441 (281,302–631,073)	3,187.51 (1,972.77–4,426.64)	−3.26 (−3.58 to −2.95)
Central Asia	3,405,798 (2,482,219–4,045,162)	9,710.49 (7,077.2–11,533.69)	505,221 (345,446–685,844)	1,372.38 (938.38–1,863.04)	−6.11 (−6.5 to −5.73)
Central Europe	478,669 (344,104–593,839)	1,422.88 (1,022.85–1,765.17)	28,820 (17,723–40,485)	139.78 (85.86–196.44)	−6.74 (−7.03 to −6.45)
Central Latin America	5,495,756 (4,008,013–6,537,654)	6,487.47 (4,732.19–7,716.85)	543,288 (358,686–760,283)	728.21 (479.37–1,020.76)	−6.26 (−6.47 to −6.05)
Central Sub-Saharan Africa	13,176,857 (6,613,548–17,564,428)	34,506.36 (17,329.22–46,000.14)	3,762,190 (614,500–6,893,669)	4,852.77 (794.5–8,890.4)	−6.23 (−6.84 to −5.6)
East Asia	25,323,758 (18,438,436–31,001,525)	5,938.79 (4,323.86–7,270.4)	485,721 (319,877–723,777)	164.06 (107.94–244.63)	−12.75 (−13.6 to −11.88)
Eastern Europe	541,338 (394,730–667,498)	851.6 (621.01–1,049.87)	42,952 (30,307–56,936)	113.82 (80.42–150.59)	−6.56 (−7.05 to −6.06)
Eastern Sub-Saharan Africa	49,176,441 (33,344,009–59,246,169)	37,093.17 (25,159.63–44,695.72)	13,384,124 (7,008,999–19,651,727)	5,701.1 (2,986.92–8,369.61)	−6.03 (−6.34 to −5.71)
High-income Asia Pacific	63,546 (40,267–86,069)	167.97 (106.34–227.49)	7,021 (2,524–11,576)	29.22 (10.35–48.29)	−4.5 (−4.81 to −4.19)
High-income North America	69,698 (39,142–113,172)	87.09 (48.98–141.14)	33,098 (18,692–53,194)	39.29 (22.31–63.07)	−1.48 (−1.81 to −1.16)
North Africa and Middle East	17,347,591 (12,906,941–21,872,134)	9,198.1 (6,843.75–11,597.13)	2,285,124 (1,547,622–2,946,579)	1,013.84 (686.52–1,307.33)	−6.82 (−7.1 to −6.55)
Oceania	510,617 (375,336–653,007)	13,811.04 (10,151.92–17,662.67)	419,666 (279,268–567,641)	5,893.55 (3,921.99–7,971.72)	−1.93 (−2.22 to −1.63)
South Asia	110,192,538 (89,209,447–126,897,262)	19,061.31 (15,431.04–21,952.05)	14,360,389 (10,551,864–18,360,346)	2,451.5 (1,801.75–3,133.28)	−5.87 (−6.22 to −5.52)
Southeast Asia	26,657,566 (20,196,222–31,402,822)	12,414.83 (9,405.61–14,624.45)	2,682,059 (1,794,366–3,465,196)	1,290.75 (863.01–1,667.89)	−6.75 (−6.87 to −6.64)
Southern Latin America	230,385 (170,454–287,333)	1,214.95 (898.84–1,515.32)	17,251 (11,176–25,178)	107.29 (69.3–156.72)	−6.71 (−7.12 to −6.29)
Southern Sub-Saharan Africa	3,564,254 (2,557,679–4,233,884)	12,955.31 (9,297.06–15,389.35)	1,320,811 (891,283–1,762,664)	4,460.6 (3,009.41–5,953.18)	−2.69 (−3.14 to −2.24)
Tropical Latin America	4,253,185 (2,796,915–5,272,938)	6,756.85 (4,442.19–8,377.52)	170,796 (110,298–238,812)	268.6 (173.22–375.82)	−9.53 (−9.81 to −9.26)
Western Europe	61,968 (33,613–98,519)	73.12 (39.65–116.11)	16,688 (5,236–32,370)	19.96 (6.33–38.4)	−2.92 (−3.23 to −2.61)
Western Sub-Saharan Africa	56,915,920 (35,081,226–70,297,317)	43,262.43 (26,669.41–53,434.83)	30,359,531 (11,730,526–44,945,183)	10,314.07 (3,986.97–15,268.21)	−4.47 (−4.77 to −4.17)

EAPC, estimated annual percentage change; SDl, Sociodemographic Index; Ul, uncertainty interval. “EAPC is expressed as 95% CIs.

**Table 3:** Burden of YLDs due to growth failure in children and adolescents (1990 and 2021).

Location	Rate per 100,000 (95%UI)				
	1990		2021		1990–2021
	YLDs cases	The age-standardized YLDs rate	YLDs cases	The age-standardized YLDs rate	EAPC
Global	3,780,864 (1,850,350–6,001,385)	165.81 (81.17–263.18)	1,901,520 (1,069,101–2,963,746)	77.4 (43.48–120.64)	−2.12 (−2.56 to −1.69)
SDI region					
High SDI	34,033 (13,638–65,367)	14.59 (5.86–27.83)	27,582 (11,456–51,248)	11.82 (4.73–22.2)	0.36 (−0.18 to 0.9)
High-middle SDI	137,732 (58,746–237,311)	39.9 (16.96–68.79)	39,746 (23,247–62,068)	14.78 (8.67–22.98)	−2.92 (−3.27 to −2.57)
Middle SDI	903,871 (449,055–1,436,488)	121.93 (60.53–193.79)	359,083 (215,572–541,669)	53.55 (32.17–80.66)	−2.17 (−2.54 to −1.8)
Low-middle SDI	1,716,184 (916,100–2,637,759)	270.86 (144.73–416.36)	723,753 (434,558–1,088,938)	101.17 (60.75–152.17)	−2.65 (−3.04 to −2.25)
Low SDI	987,499 (358,895–1,676,038)	299.73 (110.04–508.08)	750,400 (348,151–1,217,950)	123.55 (57.39–200.49)	−2.74 (−3.16 to −2.33)
GBD region					
Andean Latin America	8,352 (−6,601 to 22,991)	158.14 (−124.98 to 435.32)	1,185 (−306 to 2,925)	5.07 (−1.35 to 12.57)	−12.54 (−13.76 to −11.31)
Australasia	58 (−34 to 180)	1.01 (−0.6 to 3.16)	35 (−12 to 232)	0.49 (−0.18 to 3.1)	−0.48 (−1.04 to 0.08)
Caribbean	5,459 (1,943–10,015)	35.91 (12.78–65.87)	2,487 (1,388–3,957)	17.4 (9.72–27.66)	−2.54 (−2.93 to −2.15)
Central Asia	24,413 (12,075–40,253)	70.31 (34.8–115.95)	4,723 (2,865–7,281)	12.91 (7.82–19.92)	−6.12 (−6.66 to −5.57)
Central Europe	4,571 (2,560–7,122)	13.39 (7.51–20.83)	527 (258–899)	2.53 (1.24–4.31)	−5.71 (−6.36 to −5.07)
Central Latin America	25,465 (567–54,253)	30.11 (0.71–64.12)	4,165 (324–9,086)	5.37 (0.31–11.81)	−5.1 (−5.28 to −4.92)
Central Sub-Saharan Africa	88,651 (1,345–174,537)	233.72 (4.63–459.47)	65,732 (5,884–133,167)	84.87 (7.65–171.9)	−3.64 (−4.35 to −2.93)
East Asia	160,476 (42,836–294,617)	37.37 (9.9–68.64)	5,065 (−220 to 12,171)	1.66 (−0.08 to 3.97)	−10.17 (−10.5 to −9.83)
Eastern Europe	32,232 (17,024–53,932)	50.16 (26.49–83.93)	6,877 (4,041–10,603)	17.39 (10.3–26.63)	−3.12 (−3.58 to −2.65)
Eastern Sub-Saharan Africa	258,928 (33,541–482,788)	197.51 (26.89–367.37)	159,925 (68,583–273,758)	68.18 (29.26–116.69)	−3.7 (−4.06 to −3.34)
High-income Asia Pacific	3,533 (−701 to 8,288)	9.27 (−1.88 to 21.69)	1,811 (−585 to 4,639)	7.6 (−2.46 to 19.4)	−0.02 (−0.36 to 0.33)
High-income North America	5,299 (−845 to 17,519)	6.47 (−1.06 to 21.38)	14,896 (7,114–25,408)	15.56 (7.36–26.66)	3.3 (2.29–4.31)
North Africa and Middle East	238,935 (114,060–389,545)	127.61 (61.03–208)	93,906 (55,378–143,982)	41.46 (24.45–63.53)	−3.33 (−3.75 to −2.91)
Oceania	5,127 (760–9,820)	139.84 (21.25–267.48)	9,001 (4,554–14,010)	127.24 (64.39–198.13)	0 (−0.36 to 0.36)
South Asia	2,090,963 (1,220,597–3,117,884)	364.11 (212.58–543.13)	1,000,619 (630,708–1,456,799)	165.88 (104.76–241)	−1.87 (−2.26 to −1.49)
Southeast Asia	434,639 (219,571–684,758)	201.9 (101.94–318.09)	159,855 (90,179–243,472)	76.1 (42.89–115.9)	−2.98 (−3.24 to −2.73)
Southern Latin America	1,655 (−1,021 to 4,713)	8.73 (−5.39 to 24.86)	398 (−121 to 1,320)	2.18 (−0.77 to 7.12)	−3.76 (−4.45 to −3.07)
Southern Sub-Saharan Africa	19,552 (2,600–39,454)	71.24 (9.56–143.66)	4,690 (1,314–8,879)	15.82 (4.42–29.96)	−4.92 (−5.37 to −4.48)
Tropical Latin America	17,444 (−2,464 to 39,743)	27.4 (−4.07 to 62.61)	2,379 (−188 to 5,749)	3.66 (−0.35 to 8.9)	−6.62 (−6.84 to −6.41)
Western Europe	4,099 (−553 to 10,794)	4.79 (−0.65 to 12.46)	6,880 (344–16,740)	7.5 (0.16–18.49)	2.64 (2.07–3.22)
Western Sub-Saharan Africa	351,012 (41,511–674,889)	269.36 (33.15–516.93)	356,366 (83,694–660,267)	121.42 (28.67–224.86)	−2.65 (−3.12 to −2.18)

EAPC, estimated annual percentage change; SDl, Sociodemographic Index; Ul, uncertainty interval. “EAPC is expressed as 95% CIs.

**Table 4:** Burden of YLLs due to growth failure in children and adolescents (1990 and 2021).

Location	Rate per 100,000 (95%UI)				
	1990		2021		1990–2021
	YLLs cases	The age-standardized YLLs rate	YLLs cases	The age-standardized YLLs rate	EAPC
Global	317,180,998 (227,994,574–371,094,523)	13,894.02 (9,987.32–16,255.81)	69,147,768 (35,230,278–97,994,188)	2,850.12 (1,451.22–4,039.78)	−4.9 (−5.21 to −4.6)
SDI region					
High SDI	581,946 (407,785–757,186)	255.74 (179.15–332.78)	55,206 (32,673–81,880)	27.57 (16.23–41.01)	−6.03 (−6.35 to −5.71)
High-middle SDI	9,108,332 (6,716,608–11,337,580)	2,660.96 (1,962.07–3,312.35)	357,952 (236,209–486,493)	138.11 (91–187.85)	−9.4 (−9.63 to −9.17)
Middle SDI	57,934,818 (42,463,641–68,794,282)	7,843.24 (5,748.53–9,313.52)	5,405,543 (3,330,377–7,368,285)	828.87 (510.03–1,130.41)	−6.61 (−6.8 to −6.43)
Low-middle SDI	124,824,621 (95,917,799–144,395,503)	19,546.37 (15,019.47–22,612.26)	18,246,676 (10,912,173–24,861,298)	2,584.25 (1,544.99–3,521.45)	−6.06 (−6.36 to −5.75)
Low SDI	124,555,272 (84,591,223–147,933,256)	37,296.1 (25,333.68–44,300.37)	45,030,813 (20,779,406–64,995,118)	7,387.85 (3,410.26–10,662.43)	−5.08 (−5.34 to −4.81)
GBD region					
Andean Latin America	1,943,013 (1,456,453–2,363,635)	9,992.73 (7,490.64–12,155.98)	168,298 (109,601–242,547)	741.01 (482.42–1,068.09)	−7.73 (−7.94 to −7.53)
Australasia	3,443 (2,183–5,452)	60.56 (38.38–95.9)	581 (341–986)	8.64 (5.07–14.69)	−3.91 (−4.51 to −3.3)
Caribbean	1,535,653 (1,081,448–1,902,305)	10,096.26 (7,110.37–12,506.93)	451,954 (279,803–628,155)	3,170.11 (1,962.3–4,406.22)	−3.27 (−3.58 to −2.95)
Central Asia	3,381,384 (2,469,799–4,009,940)	9,640.18 (7,041.35–11,432.15)	500,498 (342,200–681,603)	1,359.48 (929.52–1,851.38)	−6.11 (−6.5 to −5.73)
Central Europe	474,098 (340,941–587,657)	1,409.48 (1,013.58–1,747.11)	28,293 (17,394–39,760)	137.25 (84.29–192.96)	−6.76 (−7.05 to −6.46)
Central Latin America	5,470,290 (4,005,894–6,489,486)	6,457.35 (4,729.61–7,659.94)	539,123 (358,303–754,467)	722.84 (479.1–1,013.19)	−6.27 (−6.48 to −6.06)
Central Sub-Saharan Africa	13,088,206 (6,615,806–17,423,475)	34,272.64 (17,333.16–45,629.61)	3,696,458 (618,903–6,783,867)	4,767.9 (800.07–8,748.72)	−6.25 (−6.87 to −5.63)
East Asia	25,163,282 (18,378,614–30,827,545)	5,901.42 (4,310.05–7,229.96)	480,656 (319,107–714,809)	162.4 (107.72–241.69)	−12.77 (−13.63 to −11.9)
Eastern Europe	509,106 (377,328–621,077)	801.44 (593.96–977.73)	36,074 (24,694–48,416)	96.43 (65.92–129.53)	−6.9 (−7.42 to −6.39)
Eastern Sub-Saharan Africa	48,917,513 (33,337,690–58,867,691)	36,895.67 (25,152.19–44,408.03)	13,224,199 (6,935,550–19,389,832)	5,632.92 (2,955.55–8,258.02)	−6.05 (−6.36 to −5.73)
High-income Asia Pacific	60,013 (40,637–79,449)	158.7 (107.39–210.12)	5,210 (3,121–7,388)	21.62 (12.86–30.75)	−5.08 (−5.44 to −4.72)
High-income North America	64,399 (39,918–95,654)	80.61 (49.96–119.75)	18,202 (11,480–27,953)	23.72 (14.84–36.62)	−3.01 (−3.27 to −2.74)
North Africa and Middle East	17,108,656 (12,761,551–21,652,529)	9,070.49 (6,765.87–11,479.56)	2,191,217 (1,492,439–2,842,566)	972.38 (662.22–1,261.45)	−6.91 (−7.19 to −6.64)
Oceania	505,490 (374,766–646,140)	13,671.2 (10,135.44–17,475.67)	410,666 (273,552–557,406)	5,766.31 (3,841.06–7,826.94)	−1.95 (−2.25 to −1.66)
South Asia	108,101,575 (87,551,877–124,571,765)	18,697.2 (15,142.41–21,546.79)	13,359,771 (9,612,831–17,341,697)	2,285.63 (1,644.49–2,966.88)	−6.02 (−6.37 to −5.66)
Southeast Asia	26,222,926 (19,967,387–30,963,610)	12,212.93 (9,299.49–14,420.53)	2,522,204 (1,684,510–3,270,028)	1,214.65 (810.84–1,575.05)	−6.87 (−6.99 to −6.75)
Southern Latin America	228,729 (171,517–283,784)	1,206.22 (904.44–1,496.6)	16,853 (11,276–24,177)	105.12 (69.97–151.33)	−6.74 (−7.15 to −6.33)
Southern Sub-Saharan Africa	3,544,702 (2,556,616–4,206,633)	12,884.07 (9,293.04–15,290.12)	1,316,121 (889,083–1,755,489)	4,444.78 (3,002.01–5,928.96)	−2.68 (−3.13 to −2.23)
Tropical Latin America	4,235,741 (2,796,506–5,247,818)	6,729.45 (4,441.83–8,338.02)	168,416 (109,405–235,523)	264.94 (171.89–370.76)	−9.55 (−9.83 to −9.27)
Western Europe	57,869 (34,058–88,530)	68.33 (40.18–104.58)	9,808 (4,878–16,106)	12.46 (6.16–20.5)	−4.2 (−4.52 to −3.87)
Western Sub-Saharan Africa	56,564,908 (35,021,919–69,697,821)	42,993.07 (26,621.81–52,976.54)	30,003,164 (11,665,905–44,489,191)	10,192.65 (3,964.61–15,112.92)	−4.49 (−4.78 to −4.19)

EAPC, estimated annual percentage change; SDl, Sociodemographic Index; Ul, uncertainty interval. “EAPC is expressed as 95% CIs.

**Figure 3:**
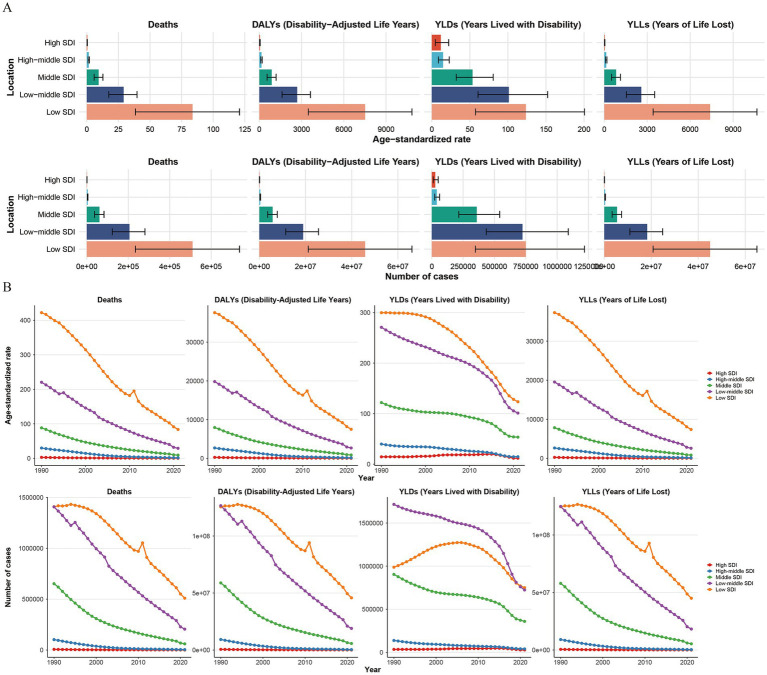
The trend changes in the number of indicators and their Age-Standardized Rates (ASRs) for the global burden of growth failure-related diseases among children and adolescents in different SDI regions from 1990 to 2021, and a comparison of the status in 2021 (**A.** Comparison of the status in 2021; **B.** Comparison of the trend changes from 1990 to 2021).

In terms of Socio-Demographic Index (SDI), the burden of disease and the ASRs for child and adolescent HF have shown a consistent decrease across all SDI regions over time. However, this burden has remained disproportionately high in lower SDI regions (Figure 3). In 2021, the number of deaths, DALYs, YLDs, and YLLs in low-SDI regions were 509,830 (95% UI: 234,467–736,295), 45,781,212 (95% UI: 21,206,233–66,073,378), 750,400 (95% UI: 348,151–1,217,950), and 45,030,813 (95% UI: 20,779,406–64,995,118), respectively. These accounted for 65.24, 64.44, 39.46, and 65.12% of the global totals, with corresponding ASRs of 83.65 (95% UI: 38.48–120.8), 7,511.4 (95% UI: 3,480.71–10,839.85), 123.55 (95% UI: 57.39–200.49), and 7,387.85 (95% UI: 3,410.26–10,662.43), respectively. These rates were 2.6, 2.57, 1.6, and 2.6 times higher than the global averages (Tables 1–4).

**Figure 4:**
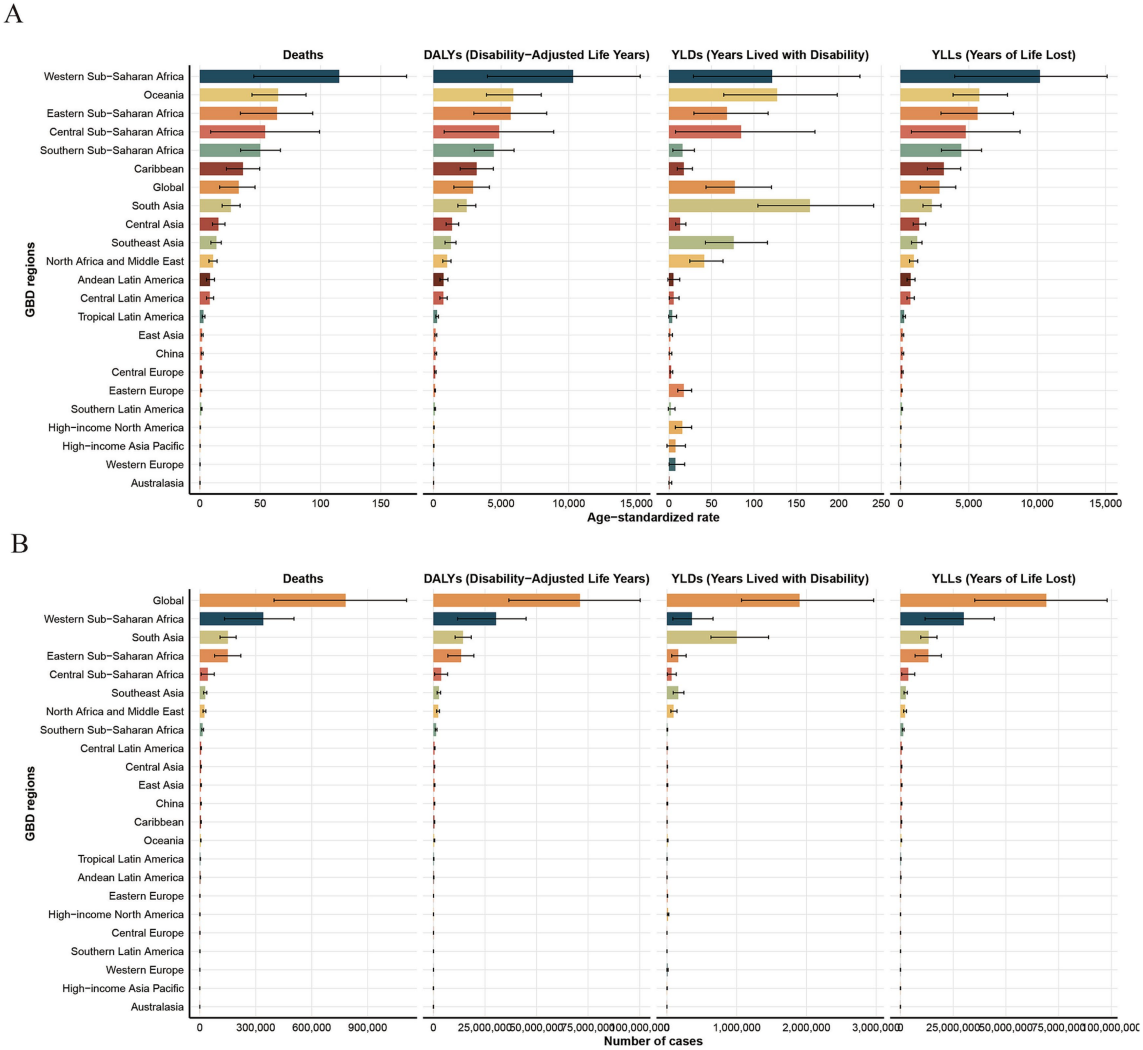
The number of indicators and their Age-Standardized Rates (ASRs) for the global burden of growth failure-related diseases among children and adolescents in different regions in 2021 (**A.** ASRs; **B.** Number of indicators).

Looking at regional trends from 1990 to 2021, the disease burden due to HF has been rapidly declining across all regions, with the most significant reductions observed in East Asia and Tropical Latin America. However, the incidence of YLDs and their ASRs has been rising in high-income regions, such as North America and Western Europe. Notably, YLDs in these regions increased by 181.1 and 67.8%, respectively, compared to 1990, with EAPCs of 3.3 (95% CI: 2.29–4.31) and 2.64 (95% CI: 2.07–3.22). The differences in ASRs between global regions are substantial, with indicators such as Age-Standardized DALYs Rates (ASDRs), Age-Standardized Mortality Rates (ASMRs), and Age-Standardized Years of Life Lost Rates (ASYLLRs) varying by over 1,000-fold. The highest rates were found in high-income regions, including Australasia, Western Europe, High-Income Asia Pacific, and North America. Furthermore, the discrepancy in Age-Standardized Years Lived with Disability Rates (ASYLDRs) was also more than 300 times higher, with South Asia and Oceania exhibiting the most extreme differences. In terms of total burden, regions like Western Sub-Saharan Africa and South Asia exhibited the highest values, collectively contributing to over 60% of the global burden across all metrics, with YLDs alone representing more than 70%. Within these regions, South Asia had the highest burden, with YLDs approximately 2.8 times greater than those in Western Sub-Saharan Africa (Figure 4 and Tables 1–4).

**Figure 5:**
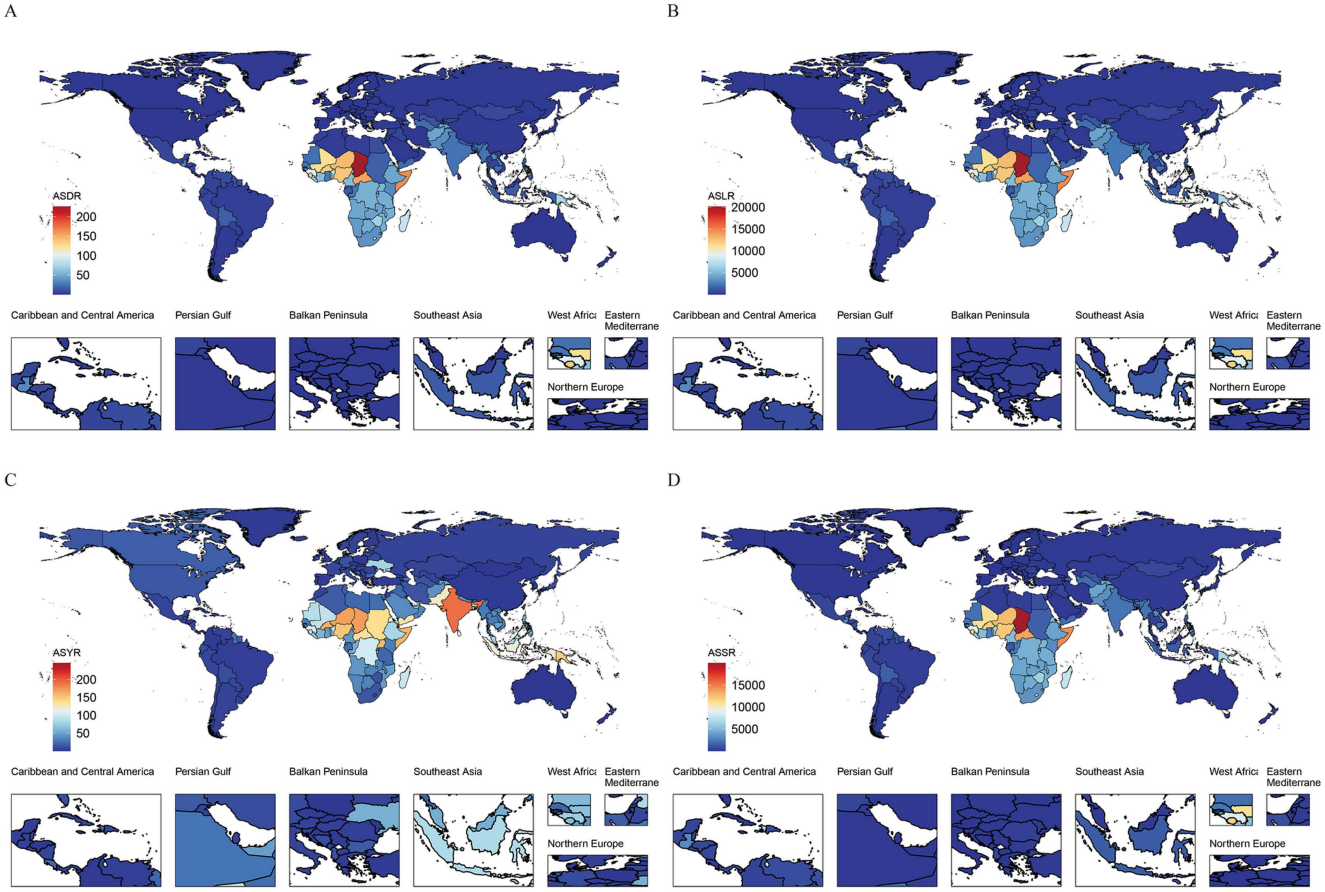
The geographical distribution of the number of indicators for the global burden of growth failure-related diseases among children and adolescents in 204 countries in 2021 (**A.** Deaths; **B.** DALYs; **C.** YLDs; **D.** YLLs).

**Figure 6:**
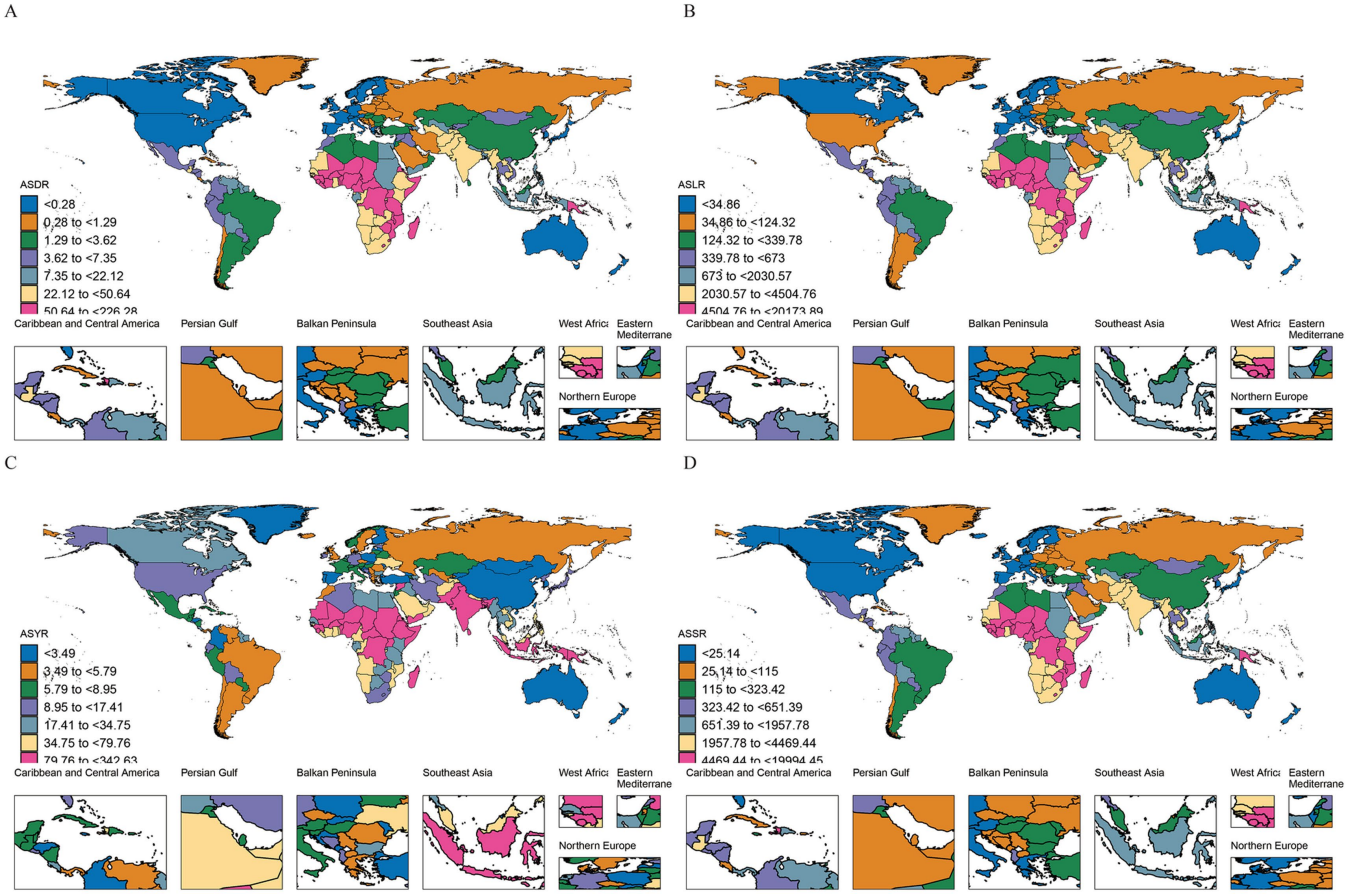
The geographical distribution of the Age-Standardized Rates (ASRs) for the global burden of growth failure-related diseases among children and adolescents in 204 countries in 2021 (**A.** Deaths; **B.** DALYs; **C.** YLDs; **D.** YLLs).

Examining trends across 204 countries, the disease burden from HF, including deaths, DALYs, YLLs, and ASRs, has been declining steadily in all countries, except Dominica and Canada. Significant declines were observed in countries such as China, the Democratic People’s Republic of Korea, and Turkey, all of which had EAPCs of ASRs greater than −10. However, the number of YLDs and their ASRs continued to increase in 26 countries, with most of these being European nations, followed by Pacific countries. Notably, Costa Rica and Canada experienced the most rapid increases in YLDs. The disparity between individual countries in terms of ASRs is far greater than regional differences. For example, the gap in ASMRs and ASYLLs can be as large as 7,500 times, such as between Chad and Andorra. Similarly, the differences in ASDRs and ASYLDRs can reach 3,000 and 1,100 times, respectively, between countries like South Sudan and Australia. Generally, countries with higher disease burdens tend to be low-income nations. For instance, Nigeria, India, and Pakistan exhibited the highest ASDRs, underscoring the critical need for targeted interventions in these regions (Figure 5, 6 and Supplementary Tables S1–S4).

### Correlation between SDI and disease burden for child and adolescent growth failure

**Figure 7:**
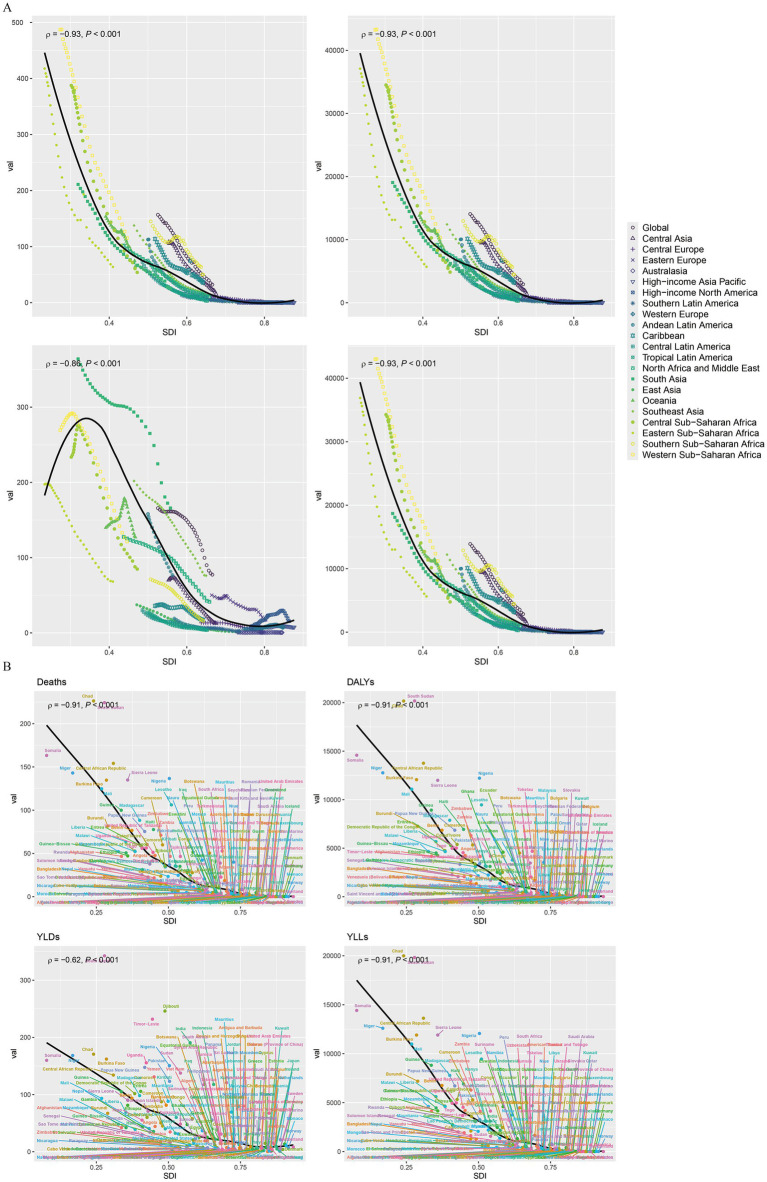
The correlation analysis between the indicators of the global burden of growth failure-related diseases among children and adolescents and SDI (**A.** Regional level; **B.** National level).

The disease burden associated with child and adolescent HF showed a significant negative correlation with the SDI at both district and national levels. Specifically, the burden decreased markedly as SDI increased, with a more pronounced effect in regions where SDI was below 0.5. At the district level, the correlation coefficients (*ρ*) for deaths, DALYs, YLDs, and YLLs were −0.93, −0.93, −0.86, and −0.93, respectively (all *p* < 0.001). At the national level, the ρ values for these indicators were −0.91, −0.62, −0.91, and −0.91, respectively, with all correlations being statistically significant (*p* < 0.001) (Figure 7).

### Factors influencing the estimated annual percentage change (EAPC)

**Figure 8:**
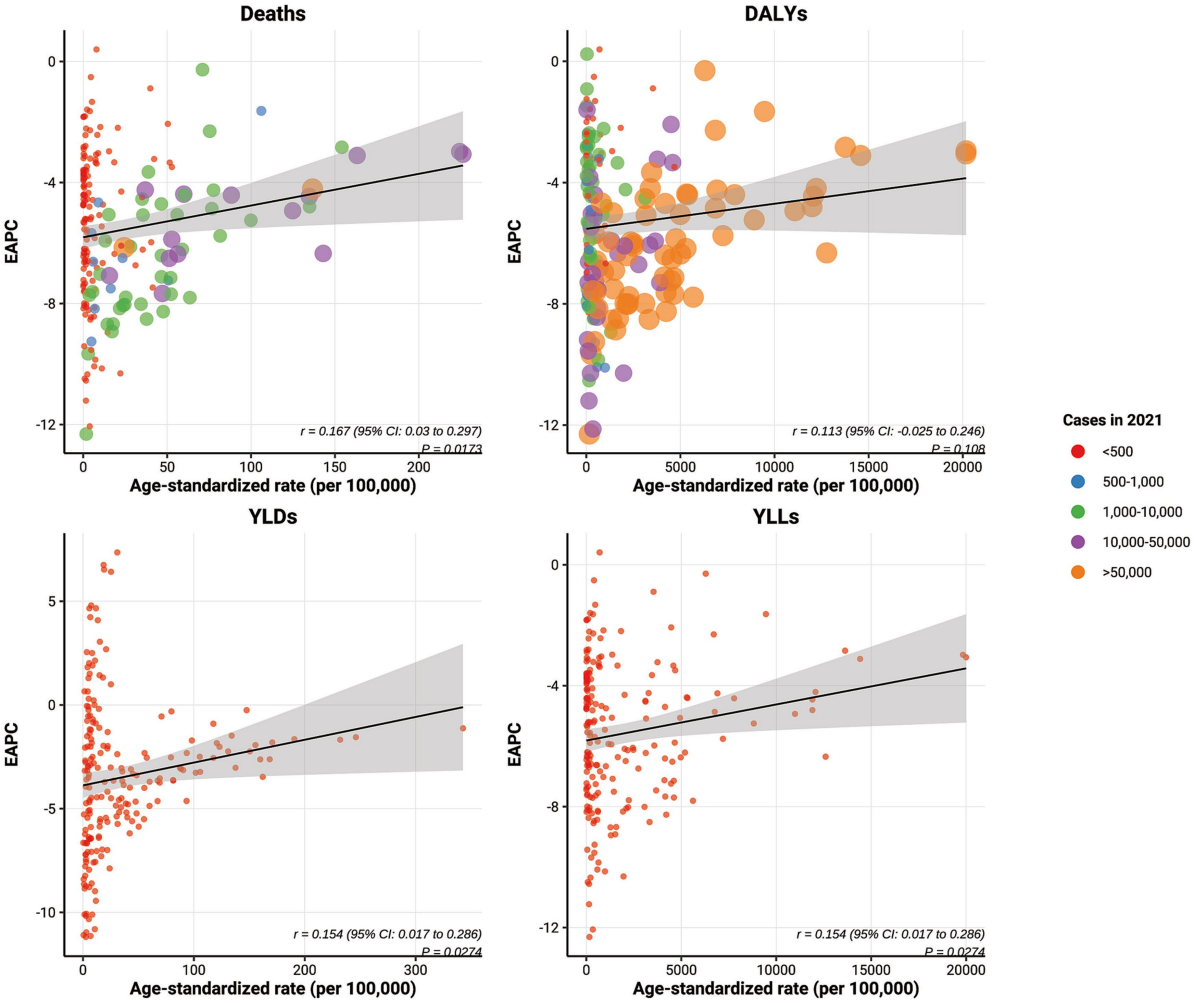
The correlation between the EAPC of the global burden of growth failure-related diseases among children and adolescents and their respective ASRs.

Figure 7 illustrates the relationship between the EAPC and ASRs for each disease burden indicator (deaths, DALYs, YLDs, and YLLs) related to child and adolescent HF on a global scale. The results reveal a general positive correlation between EAPC and SDI, indicating that as SDI increases, the EAPC for these indicators also rises. Statistically significant correlations were found for deaths, YLDs, and YLLs, with Pearson coefficients (r) of 0.167, 0.154, and 0.154, respectively (all *p* < 0.05) (Figure 8).

### Health inequities in global disease burden

**Figure 9:**
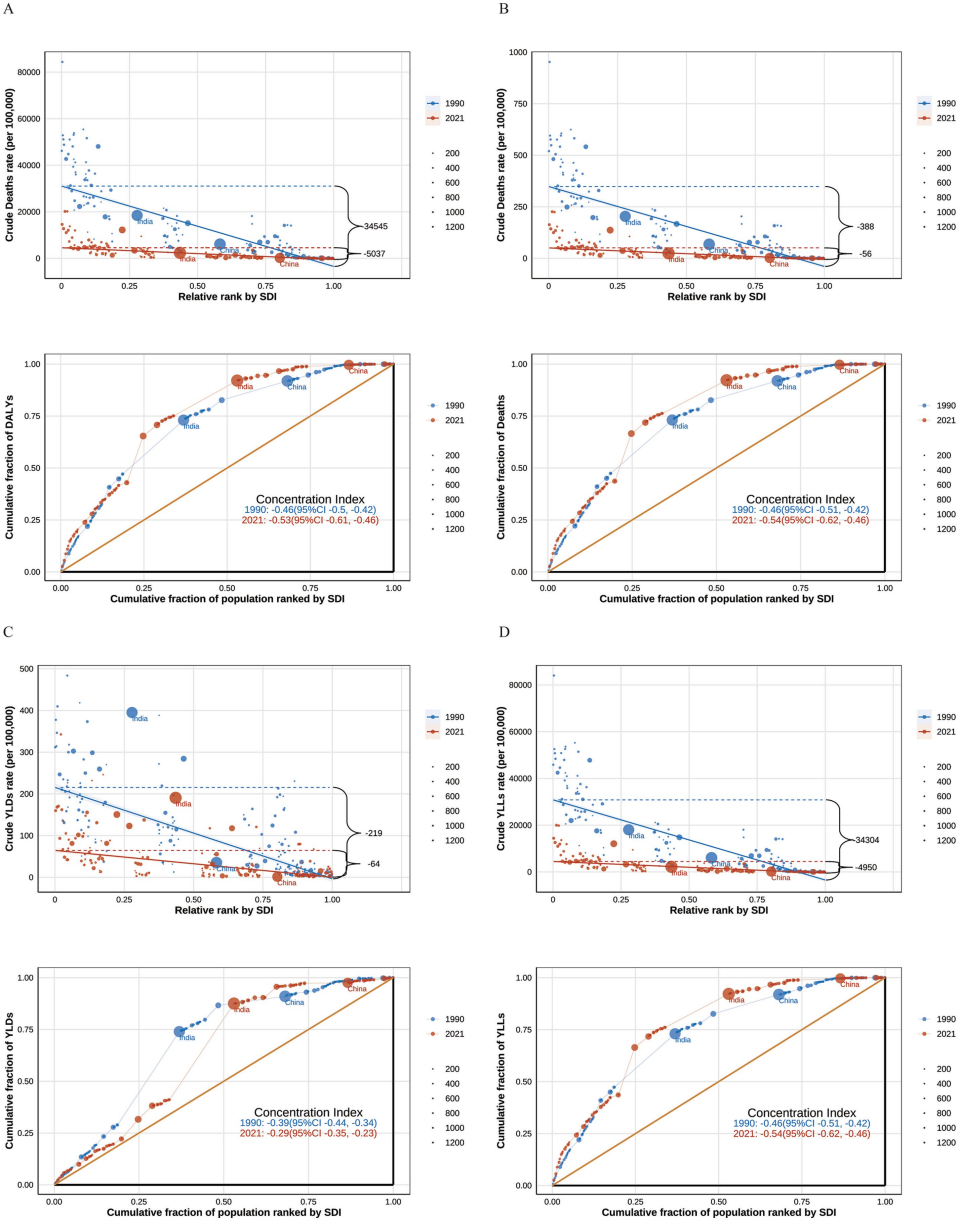
The unequal changes in the global burden of growth failure-related diseases among children and adolescents from 1990 to 2021 (**A.** Deaths; **B.** DALYs; **C.** YLDs; **D.** YLLs).

Global inequities in deaths, DALYs, YLDs, and YLLs due to child and adolescent HF persisted in 2021, compared to 1990. From 1990 to 2021, the slope indices for these indicators shifted as follows: from −387.59 to −55.99 for deaths, −34,545.31 to −5,036.63 for DALYs, −219.18 to −64.16 for YLDs, and −34,303.85 to −4,950.30 for YLLs. Correspondingly, the concentration indices for these indicators increased, from −0.46 to −0.53 for deaths, −0.46 to −0.53 for DALYs, −0.39 to −0.29 for YLDs, and −0.46 to −0.54 for YLLs. These data suggest that the global concentration of the disease burden for child and adolescent HF in low SDI regions has not improved over time (Figure 9).

### Decomposition analysis of disease burden drivers

**Figure 10:**
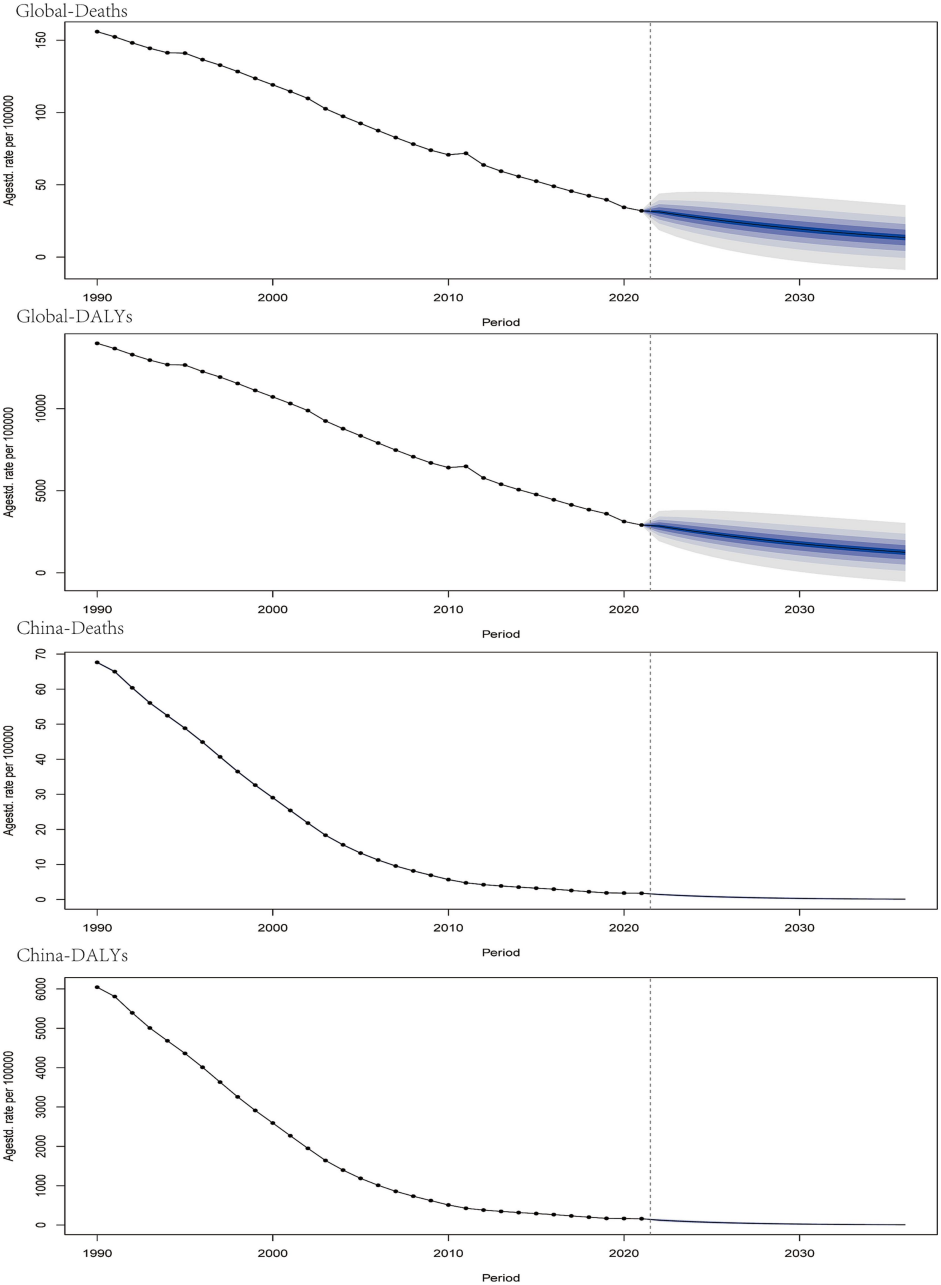
The decomposition analysis of the global burden of growth failure-related diseases among children and adolescents in terms of aging, population growth, and epidemiological changes.

To explore the underlying factors contributing to the global disease burden of child and adolescent HF, we conducted decomposition analyses, considering population growth, age distribution changes, and epidemiological shifts. The results revealed that, in low and lower-middle SDI regions, such as Africa and South Asia, population growth was the primary positive driver of the disease burden, while epidemiological changes had the most significant negative impact, followed by age distribution changes. At the global level, population growth contributed −12.43, −12.51%, −23.79%, and −12.42% to the burden of deaths, DALYs, YLDs, and YLLs, respectively, while epidemiological changes contributed 105.08, 105.12, 111.21, and 105.07% to these indicators. In contrast, in higher SDI regions such as China, Western Europe, and Eastern Europe, both population growth and age distribution changes acted as negative contributors, with epidemiological changes being the most significant negative driver (Figure 10).

### 2035 projections for child and adolescent growth failure disease burden

**Figure 11:**
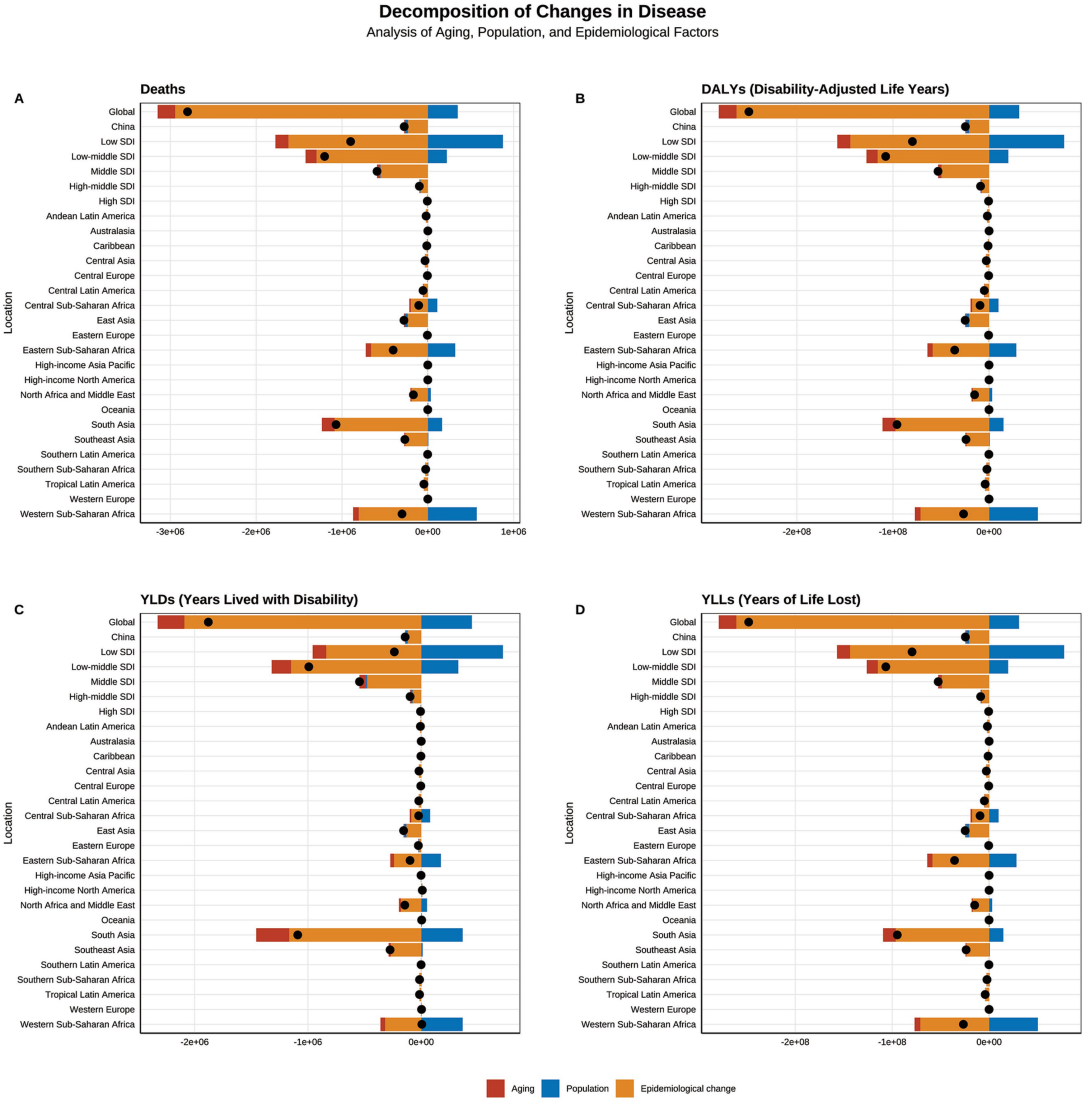
The projection of the global and Chinese burden of growth failure-related diseases among children and adolescents by 2035 (**A.** Deaths; **B.** DALYs; **C.** YLDs; **D.** YLLs).

Projections for the next decade, based on the Bayesian Annual Percentage Change (BAPC), indicate a continued decline in the global disease burden of child and adolescent HF, including deaths, DALYs, YLDs, and YLLs. In particular, China is projected to maintain its position at the lowest point of the disease burden trajectory (Figure 11).

## Discussion

To the best of our knowledge, this is the first study to assess the global and national disease burden of HF among adolescents and children, using data from the GBD database. Our findings indicate that, although the number and ASRs of the global HF-related disease burden are decreasing, the concentration of this burden in low SDI regions and among children under 5 years of age has not improved over time. This highlights the persistent and severe health disparities worldwide, underscoring the urgent need for targeted interventions in low SDI areas.

Our study aligns with previous research indicating that HF in adolescents and children is predominantly concentrated in children under five, particularly in low- and middle-income regions, such as Africa, South Asia, and Southeast Asia (16, 21, 22). Specifically, Southeast Asia and Sub-Saharan Africa account for 90% of underweight children. Sub-Saharan Africa, in particular, is the only region where the number of children experiencing stunting has increased, with an approximate one-third rise between 1990 and 2013. Moreover, this region accounts for one-third of the global total of children with HF. In contrast, our study reveals that low SDI regions alone account for over 65% of global deaths, DALYs, and YLLs, with Western Sub-Saharan Africa and South Asia contributing 60% of these burdens. Notably, the number of YLDs in these regions exceeds 70%. Nigeria, India, and Pakistan are the most affected countries. These regions also exhibit the highest ASRs globally, with discrepancies as large as 1,000 times compared to the lowest regions. Chad, in particular, shows an alarming difference of up to 7,500 times compared to Andorra. Furthermore, our study highlights that the vast majority of deaths and DALYs associated with HF occur in children under 5 years of age, representing more than 98% of cases. The corresponding ASRs in this age group are also significantly higher—over 200 times greater than in other age groups. These findings emphasize the exceptionally severe disease burden among children in the regions and countries mentioned above. More concerning, our decomposition analysis reveals that from 1990 to 2021, these regions have not implemented effective interventions to address childhood HF, despite population growth. Consequently, the trend of high mortality and high DALY concentration remains unchanged. In contrast, East Asian countries such as China and the Democratic People’s Republic of Korea have demonstrated significant progress, with rapid declines in their corresponding ASRs. This underscores the positive impact of these countries’ efforts to address childhood HF.

At the global level, our study found that males consistently exhibit higher rates of HF and ASRs compared to females, a pattern that aligns with previous research (23–25). To further explore gender differences in childhood HF, Thurstans et al. recently conducted a pooled analysis of 77 studies. Their findings suggest that the sex ratio of children affected by HF varies by age and geographic location. Specifically, boys under 30 months of age are more susceptible to HF, but this trend diminishes after 30 months. Moreover, globally—particularly in Africa—boys are almost always at greater risk of HF than girls. However, in South Asia and Central America, the odds ratios (ORs) for underweight and wasting in boys have declined, and in some cases, even reversed (23). Despite these observed patterns, most studies included in the analysis did not investigate the underlying causes of gender disparities in HF. Limited available explanations suggest that boys may be more biologically vulnerable than girls from conception onward. Common childhood illnesses, such as respiratory infections, diarrhea, malaria, and prematurity, are generally more prevalent in boys, increasing their risk of mortality as well as their likelihood of experiencing weight loss, developmental delays, or severe HF (26, 27). Additionally, sex hormones—including testosterone, luteinizing hormone, and follicle-stimulating hormone—may contribute to these differences (28). Geographic variations in gender disparities may also be influenced by differences in parental caregiving behaviors, which are shaped by cultural norms, particularly in patriarchal societies (14, 23, 29, 30). However, these explanations remain largely speculative, as they are based more on assumptions about social factors than on conclusive empirical evidence. Future research should prioritize investigating the biological and sociocultural mechanisms contributing to gender differences in childhood HF to inform more targeted interventions.

In our study, we observed a rapid decline in the global burden of disease related to HF among children and adolescents, as well as their ASRs, over the past 30 years across all regions and age groups. However, despite these improvements, the current status remains unacceptable and still falls short of the World Health Assembly’s goal of reducing stunting rates by 40% by 2025 and maintaining the prevalence of wasting below 5% (31). Notably, ASRs in countries with predominantly high national disease burdens, such as Costa Rica and Canada, continue to show rapid growth. Over the decades, multiple factors influencing childhood HF have been identified, including insufficient food intake, diarrhea, recurrent infections, poor hygiene practices, inappropriate feeding practices, low parental education, and low maternal BMI (1, 16, 21). Addressing the current disease burden of childhood HF requires a comprehensive and multifaceted intervention approach. At the global level, international humanitarian assistance should be directed to low SDI regions, particularly in Western Sub-Saharan Africa and South Asia. At the national level, improving the material conditions of low-income families is essential to ensuring a consistent and safe nutritional supply. Public water and sanitation systems should be improved or expanded, and parental education, particularly maternal education, should be prioritized (19). At the health system level, special attention should be given to the critical period of brain development and linear growth during the first 1,000 days post-conception, during which adequate nutrition for both mother and infant is crucial (21). Scientific therapeutic feeding practices should also be promoted, including prenatal micronutrient supplementation, exclusive breastfeeding for the first 6 months, followed by the gradual introduction of nutritionally adequate complementary solid foods, and continued breastfeeding until 2 years of age or longer (19, 32–34). Moreover, stunting, wasting, and underweight should be treated as integrated aspects of nutritional intervention rather than separate issues (1).

Our study offers a global overview of the disease burden related to HF in children and adolescents across 21 super-regions and 204 countries. It compares these findings with previous studies and examines variations across regions, countries, age groups, and gender. This analysis enhances our understanding of the global burden of disease associated with HF.

Despite the important findings of this study, its limitations remain non-negligible, and the results should be interpreted with caution. Our analyses rely on finalized GBD 2021 models (e.g., DisMod-MR, cause redistribution, CodCorrect), so potential biases from data sparsity, representativeness, misclassification, and modeling assumptions—particularly in low-resource settings—may persist. Growth failure was defined using WHO anthropometry (stunting, wasting, underweight), and cross-walks (e.g., MUAC → WHZ), age extensions beyond under-5 s, survey variability, and measurement error may reduce comparability and increase uncertainty (35, 36). We report burden outcomes (deaths, YLLs, YLDs, DALYs) without re-estimating incidence or prevalence, and overlapping phenotypes and comorbidities may influence YLD estimates despite uncertainty propagation. Inequality and decomposition analyses are descriptive and ecological, such that unmeasured factors (e.g., WASH, food prices, conflict, pandemics, climate shocks) may confound trends; projections are trend-based baselines rather than causal forecasts. Finally, although we observe a higher burden in boys and in children under 5, biological versus sociocultural pathways cannot be disentangled in this analysis.

In conclusion, from 1990 to 2021, the overall burden of growth failure among children and adolescents declined substantially, with especially pronounced reductions in China; however, low-SDI regions—particularly Western Sub-Saharan Africa and South Asia—and children under 5 years of age remain the primary high-burden groups. Boys consistently bear a higher burden than girls, suggesting the influence of multiple biological and social mechanisms. We recommend prioritizing the “first 1,000 days” window in clinical practice, strengthening routine growth monitoring, and implementing early interventions. Policy-making should increase resource allocation to high-burden areas, integrate nutrition services with maternal–child health and primary care systems, and enhance data quality and tracking. In terms of research, further work is needed to elucidate the mechanisms underlying sex and age differences, optimize monitoring indicators and quantitative tools, and evaluate the effectiveness of multicomponent intervention packages.

## Data Availability

The original contributions presented in the study are included in the article/Supplementary material, further inquiries can be directed to the corresponding author.

## Footnotes

^1^ http://ghdx.healthdata.org/
