# Supplementary material for: Global and Chinese growth failure disease burden analysis and projections for adolescents and children, 1990–2021
Source: Front Public Health. 2025 Oct 9;13:1639801. doi: 10.3389/fpubh.2025.1639801 (PMC12545010; doi:10.3389/fpubh.2025.1639801)
Supplement: Supplementary file 1 [file Data_Sheet_1.doc]

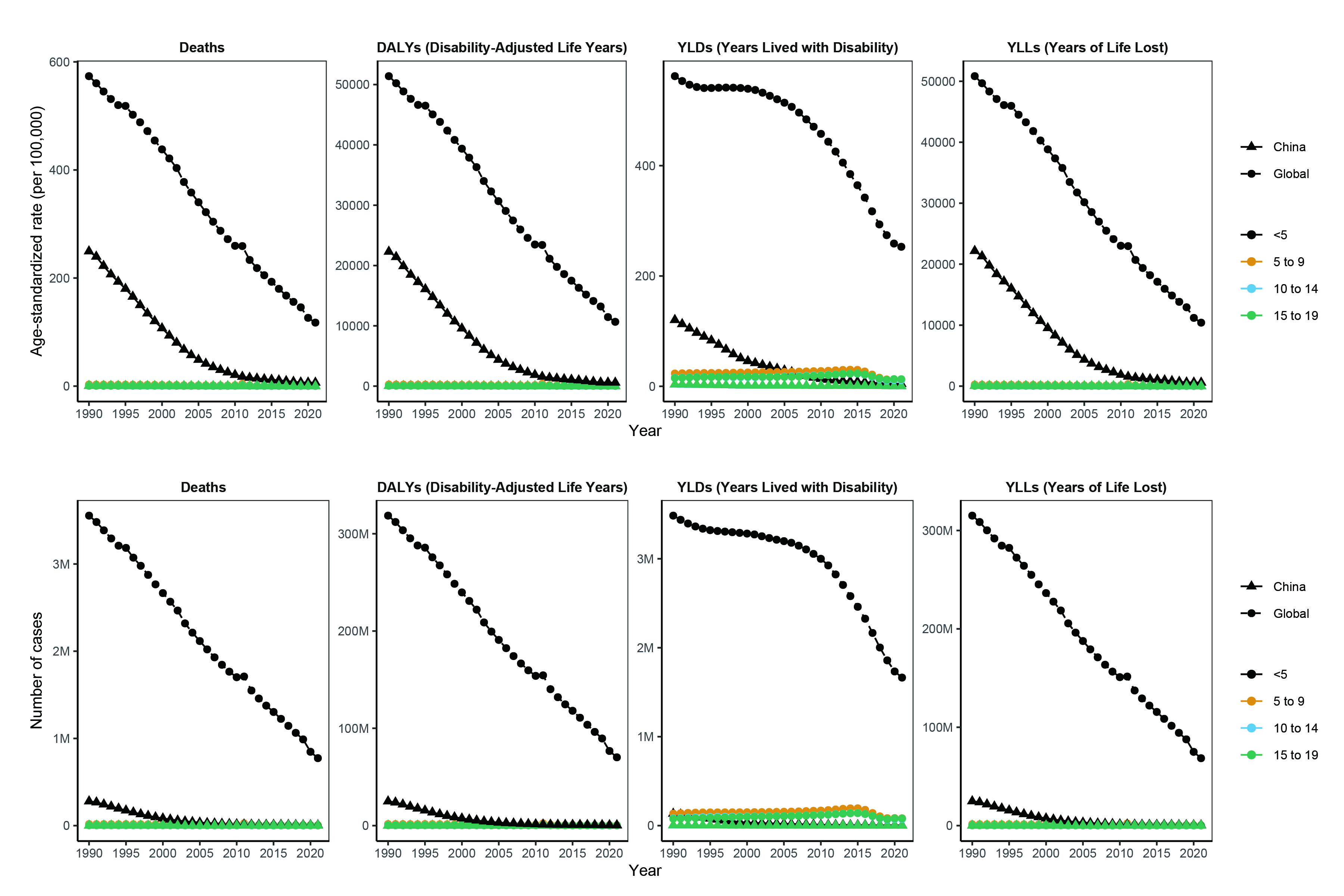


**Fig.S1** The trend changes in the number of indicators and their Age-Standardized Rates (ASRs) for the global and Chinese burden of growth failure-related diseases among children and adolescents of different age groups from 1990 to 2021.
