# Supplementary material for: Global and Chinese growth failure disease burden analysis and projections for adolescents and children, 1990–2021
Source: Front Public Health. 2025 Oct 9;13:1639801. doi: 10.3389/fpubh.2025.1639801 (PMC12545010; doi:10.3389/fpubh.2025.1639801)
Supplement: Supplementary file 2 [file Data_Sheet_2.docx]

Table.1 The deaths cases and age-standardized deaths rate of growth failure in children and adolescents in 1990 and 2021, along with their temporal trend.

| **Location** | Rate per 100 000(95%UI) | | 2021 | | 1990-2021 |
| --- | --- | --- | --- | --- | --- |
|  | 1990 | |  | |  |
|  | Deaths cases | The age-standardized deaths rate | Deaths cases | The age-standardized deaths rate | EAPC |

| **Global** | 3580631 (2574094 to 4189794) | 156.85 (112.76 to 183.54) | 781528 (397299 to 1108644) | 32.21 (16.36 to 45.7) | -4.9 (-5.2 to -4.6) |
| --- | --- | --- | --- | --- | --- |
| **SDI region** |  |  |  |  |  |
| High SDI | 6549 (4592 to 8523) | 2.88 (2.02 to 3.75) | 624 (371 to 924) | 0.31 (0.18 to 0.46) | -6.03 (-6.34 to -5.71) |
| High-middle SDI | 102501 (75619 to 127529) | 29.94 (22.09 to 37.26) | 4032 (2664 to 5477) | 1.55 (1.03 to 2.11) | -9.4 (-9.63 to -9.17) |
| Middle SDI | 652274 (478257 to 774448) | 88.3 (64.74 to 104.84) | 60887 (37498 to 83008) | 9.33 (5.74 to 12.73) | -6.62 (-6.8 to -6.43) |
| Low-middle SDI | 1407916 (1081317 to 1629239) | 220.48 (169.33 to 255.15) | 205574 (122880 to 280325) | 29.11 (17.4 to 39.7) | -6.06 (-6.37 to -5.76) |
| Low SDI | 1409408 (956870 to 1674451) | 422.09 (286.62 to 501.52) | 509830 (234467 to 736295) | 83.65 (38.48 to 120.8) | -5.08 (-5.34 to -4.81) |
| **GBD region** |  |  |  |  |  |
| Andean Latin America | 21892 (16420 to 26634) | 112.59 (84.45 to 136.98) | 1901 (1240 to 2737) | 8.37 (5.46 to 12.05) | -7.73 (-7.93 to -7.53) |
| Australasia | 39 (25 to 61) | 0.68 (0.43 to 1.08) | 7 (4 to 11) | 0.1 (0.06 to 0.17) | -3.9 (-4.51 to -3.3) |
| Caribbean | 17283 (12186 to 21424) | 113.63 (80.12 to 140.86) | 5083 (3147 to 7065) | 35.65 (22.06 to 49.55) | -3.27 (-3.59 to -2.95) |
| Central Asia | 38007 (27768 to 45079) | 108.36 (79.17 to 128.52) | 5624 (3846 to 7659) | 15.28 (10.45 to 20.81) | -6.12 (-6.5 to -5.73) |
| Central Europe | 5313 (3823 to 6587) | 15.8 (11.36 to 19.58) | 318 (195 to 446) | 1.54 (0.95 to 2.16) | -6.75 (-7.05 to -6.46) |
| Central Latin America | 61571 (45141 to 73023) | 72.69 (53.3 to 86.2) | 6096 (4063 to 8516) | 8.16 (5.42 to 11.42) | -6.26 (-6.47 to -6.05) |
| Central Sub-Saharan Africa | 147950 (74639 to 196956) | 387.49 (195.6 to 515.9) | 41881 (6923 to 76947) | 54.03 (8.95 to 99.24) | -6.25 (-6.87 to -5.62) |
| East Asia | 283153 (206849 to 346897) | 66.4 (48.51 to 81.35) | 5401 (3588 to 8029) | 1.82 (1.21 to 2.71) | -12.77 (-13.63 to -11.9) |
| Eastern Europe | 5715 (4236 to 6973) | 9 (6.67 to 10.98) | 405 (278 to 543) | 1.08 (0.74 to 1.45) | -6.9 (-7.41 to -6.38) |
| Eastern Sub-Saharan Africa | 553622 (377382 to 666081) | 417.7 (284.83 to 502.64) | 149807 (78674 to 219596) | 63.82 (33.53 to 93.53) | -6.04 (-6.36 to -5.72) |
| High-income Asia Pacific | 677 (459 to 896) | 1.79 (1.21 to 2.37) | 59 (36 to 84) | 0.24 (0.15 to 0.35) | -5.07 (-5.43 to -4.71) |
| High-income North America | 722 (448 to 1073) | 0.9 (0.56 to 1.34) | 207 (131 to 316) | 0.27 (0.17 to 0.41) | -2.99 (-3.25 to -2.73) |
| North Africa and Middle East | 192693 (143803 to 243867) | 102.16 (76.24 to 129.3) | 24680 (16817 to 32034) | 10.95 (7.46 to 14.21) | -6.91 (-7.19 to -6.64) |
| Oceania | 5682 (4214 to 7264) | 153.68 (113.96 to 196.46) | 4615 (3073 to 6268) | 64.8 (43.15 to 88.02) | -1.95 (-2.25 to -1.66) |
| South Asia | 1219248 (987375 to 1405425) | 210.89 (170.78 to 243.1) | 150131 (108038 to 194820) | 25.68 (18.48 to 33.33) | -6.03 (-6.38 to -5.67) |
| Southeast Asia | 295782 (225379 to 349325) | 137.75 (104.96 to 162.69) | 28410 (19002 to 36825) | 13.68 (9.14 to 17.73) | -6.88 (-7 to -6.76) |
| Southern Latin America | 2566 (1926 to 3183) | 13.53 (10.15 to 16.79) | 191 (128 to 274) | 1.19 (0.79 to 1.71) | -6.72 (-7.13 to -6.31) |
| Southern Sub-Saharan Africa | 39888 (28791 to 47341) | 144.99 (104.65 to 172.08) | 14825 (10017 to 19769) | 50.06 (33.82 to 66.76) | -2.67 (-3.12 to -2.23) |
| Tropical Latin America | 47447 (31339 to 58776) | 75.37 (49.77 to 93.38) | 1903 (1240 to 2657) | 2.99 (1.95 to 4.18) | -9.53 (-9.81 to -9.25) |
| Western Europe | 650 (383 to 994) | 0.77 (0.45 to 1.17) | 111 (55 to 181) | 0.14 (0.07 to 0.23) | -4.19 (-4.51 to -3.87) |
| Western Sub-Saharan Africa | 640730 (396381 to 789657) | 487.03 (301.33 to 600.25) | 339874 (131653 to 504460) | 115.47 (44.75 to 171.37) | -4.49 (-4.78 to -4.19) |

Abbreviations: EAPC, estimated annual percentage change, SDl, Sociodemographic Index; Ul,uncertainty interval. “ EAPC is expressed as 95% CIs.

Table.2 The DALYs cases and age-standardized DALYs rate of growth failure in children and adolescents in 1990 and 2021, along with their temporal trend.

| **Location** | Rate per 100 000(95%UI) | | 2021 | | 1990-2021 |
| --- | --- | --- | --- | --- | --- |
|  | 1990 | |  | |  |
|  | DALYs cases | The age-standardized DALYs rate | DALYs cases | The age-standardized DALYs rate | EAPC |

| **Global** | 320961861 (229949302 to 375935595) | 14059.83 (10073.14 to 16468.1) | 71049289 (36584453 to 100207771) | 2927.51 (1506.27 to 4129.64) | -4.85 (-5.16 to -4.55) |
| --- | --- | --- | --- | --- | --- |
| **SDI region** |  |  |  |  |  |
| High SDI | 615979 (425893 to 810318) | 270.33 (186.94 to 355.29) | 82789 (44372 to 131318) | 39.39 (21.06 to 62.31) | -5.04 (-5.36 to -4.72) |
| High-middle SDI | 9246064 (6787006 to 11501112) | 2700.86 (1982.37 to 3359.69) | 397697 (263370 to 543198) | 152.89 (101.13 to 208.85) | -9.14 (-9.36 to -8.92) |
| Middle SDI | 58838689 (42975266 to 69697281) | 7965.17 (5817.47 to 9435.15) | 5764626 (3606997 to 7797428) | 882.42 (551.42 to 1193.98) | -6.48 (-6.66 to -6.29) |
| Low-middle SDI | 126540805 (97243327 to 146238113) | 19817.23 (15228.83 to 22903.32) | 18970429 (11429673 to 25649639) | 2685.42 (1617.33 to 3631.2) | -5.98 (-6.28 to -5.68) |
| Low SDI | 125542771 (85043371 to 149306509) | 37595.83 (25473.23 to 44716.59) | 45781212 (21206233 to 66073378) | 7511.4 (3480.71 to 10839.85) | -5.05 (-5.32 to -4.79) |
| **GBD region** |  |  |  |  |  |
| Andean Latin America | 1951365 (1448797 to 2378224) | 10035.67 (7451.28 to 12230.98) | 169483 (109546 to 244332) | 746.09 (482.09 to 1075.78) | -7.73 (-7.93 to -7.53) |
| Australasia | 3501 (2146 to 5605) | 61.57 (37.72 to 98.6) | 616 (325 to 1209) | 9.13 (4.83 to 17.67) | -3.83 (-4.42 to -3.23) |
| Caribbean | 1541112 (1083763 to 1907913) | 10132.16 (7125.62 to 12543.81) | 454441 (281302 to 631073) | 3187.51 (1972.77 to 4426.64) | -3.26 (-3.58 to -2.95) |
| Central Asia | 3405798 (2482219 to 4045162) | 9710.49 (7077.2 to 11533.69) | 505221 (345446 to 685844) | 1372.38 (938.38 to 1863.04) | -6.11 (-6.5 to -5.73) |
| Central Europe | 478669 (344104 to 593839) | 1422.88 (1022.85 to 1765.17) | 28820 (17723 to 40485) | 139.78 (85.86 to 196.44) | -6.74 (-7.03 to -6.45) |
| Central Latin America | 5495756 (4008013 to 6537654) | 6487.47 (4732.19 to 7716.85) | 543288 (358686 to 760283) | 728.21 (479.37 to 1020.76) | -6.26 (-6.47 to -6.05) |
| Central Sub-Saharan Africa | 13176857 (6613548 to 17564428) | 34506.36 (17329.22 to 46000.14) | 3762190 (614500 to 6893669) | 4852.77 (794.5 to 8890.4) | -6.23 (-6.84 to -5.6) |
| East Asia | 25323758 (18438436 to 31001525) | 5938.79 (4323.86 to 7270.4) | 485721 (319877 to 723777) | 164.06 (107.94 to 244.63) | -12.75 (-13.6 to -11.88) |
| Eastern Europe | 541338 (394730 to 667498) | 851.6 (621.01 to 1049.87) | 42952 (30307 to 56936) | 113.82 (80.42 to 150.59) | -6.56 (-7.05 to -6.06) |
| Eastern Sub-Saharan Africa | 49176441 (33344009 to 59246169) | 37093.17 (25159.63 to 44695.72) | 13384124 (7008999 to 19651727) | 5701.1 (2986.92 to 8369.61) | -6.03 (-6.34 to -5.71) |
| High-income Asia Pacific | 63546 (40267 to 86069) | 167.97 (106.34 to 227.49) | 7021 (2524 to 11576) | 29.22 (10.35 to 48.29) | -4.5 (-4.81 to -4.19) |
| High-income North America | 69698 (39142 to 113172) | 87.09 (48.98 to 141.14) | 33098 (18692 to 53194) | 39.29 (22.31 to 63.07) | -1.48 (-1.81 to -1.16) |
| North Africa and Middle East | 17347591 (12906941 to 21872134) | 9198.1 (6843.75 to 11597.13) | 2285124 (1547622 to 2946579) | 1013.84 (686.52 to 1307.33) | -6.82 (-7.1 to -6.55) |
| Oceania | 510617 (375336 to 653007) | 13811.04 (10151.92 to 17662.67) | 419666 (279268 to 567641) | 5893.55 (3921.99 to 7971.72) | -1.93 (-2.22 to -1.63) |
| South Asia | 110192538 (89209447 to 126897262) | 19061.31 (15431.04 to 21952.05) | 14360389 (10551864 to 18360346) | 2451.5 (1801.75 to 3133.28) | -5.87 (-6.22 to -5.52) |
| Southeast Asia | 26657566 (20196222 to 31402822) | 12414.83 (9405.61 to 14624.45) | 2682059 (1794366 to 3465196) | 1290.75 (863.01 to 1667.89) | -6.75 (-6.87 to -6.64) |
| Southern Latin America | 230385 (170454 to 287333) | 1214.95 (898.84 to 1515.32) | 17251 (11176 to 25178) | 107.29 (69.3 to 156.72) | -6.71 (-7.12 to -6.29) |
| Southern Sub-Saharan Africa | 3564254 (2557679 to 4233884) | 12955.31 (9297.06 to 15389.35) | 1320811 (891283 to 1762664) | 4460.6 (3009.41 to 5953.18) | -2.69 (-3.14 to -2.24) |
| Tropical Latin America | 4253185 (2796915 to 5272938) | 6756.85 (4442.19 to 8377.52) | 170796 (110298 to 238812) | 268.6 (173.22 to 375.82) | -9.53 (-9.81 to -9.26) |
| Western Europe | 61968 (33613 to 98519) | 73.12 (39.65 to 116.11) | 16688 (5236 to 32370) | 19.96 (6.33 to 38.4) | -2.92 (-3.23 to -2.61) |
| Western Sub-Saharan Africa | 56915920 (35081226 to 70297317) | 43262.43 (26669.41 to 53434.83) | 30359531 (11730526 to 44945183) | 10314.07 (3986.97 to 15268.21) | -4.47 (-4.77 to -4.17) |

Abbreviations: EAPC, estimated annual percentage change, SDl, Sociodemographic Index; Ul,uncertainty interval. “ EAPC is expressed as 95% CIs.

Table.3 The YLDs cases and age-standardized YLDs rate of growth failure in children and adolescents in 1990 and 2021, along with their temporal trend.

| **Location** | Rate per 100 000(95%UI) | | 2021 | | 1990-2021 |
| --- | --- | --- | --- | --- | --- |
|  | 1990 | |  | |  |
|  | YLDs cases | The age-standardized YLDs rate | YLDs cases | The age-standardized YLDs rate | EAPC |

| **Global** | 3780864 (1850350 to 6001385) | 165.81 (81.17 to 263.18) | 1901520 (1069101 to 2963746) | 77.4 (43.48 to 120.64) | -2.12 (-2.56 to -1.69) |
| --- | --- | --- | --- | --- | --- |
| **SDI region** |  |  |  |  |  |
| High SDI | 34033 (13638 to 65367) | 14.59 (5.86 to 27.83) | 27582 (11456 to 51248) | 11.82 (4.73 to 22.2) | 0.36 (-0.18 to 0.9) |
| High-middle SDI | 137732 (58746 to 237311) | 39.9 (16.96 to 68.79) | 39746 (23247 to 62068) | 14.78 (8.67 to 22.98) | -2.92 (-3.27 to -2.57) |
| Middle SDI | 903871 (449055 to 1436488) | 121.93 (60.53 to 193.79) | 359083 (215572 to 541669) | 53.55 (32.17 to 80.66) | -2.17 (-2.54 to -1.8) |
| Low-middle SDI | 1716184 (916100 to 2637759) | 270.86 (144.73 to 416.36) | 723753 (434558 to 1088938) | 101.17 (60.75 to 152.17) | -2.65 (-3.04 to -2.25) |
| Low SDI | 987499 (358895 to 1676038) | 299.73 (110.04 to 508.08) | 750400 (348151 to 1217950) | 123.55 (57.39 to 200.49) | -2.74 (-3.16 to -2.33) |
| **GBD region** |  |  |  |  |  |
| Andean Latin America | 8352 (-6601 to 22991) | 158.14 (-124.98 to 435.32) | 1185 (-306 to 2925) | 5.07 (-1.35 to 12.57) | -12.54 (-13.76 to -11.31) |
| Australasia | 58 (-34 to 180) | 1.01 (-0.6 to 3.16) | 35 (-12 to 232) | 0.49 (-0.18 to 3.1) | -0.48 (-1.04 to 0.08) |
| Caribbean | 5459 (1943 to 10015) | 35.91 (12.78 to 65.87) | 2487 (1388 to 3957) | 17.4 (9.72 to 27.66) | -2.54 (-2.93 to -2.15) |
| Central Asia | 24413 (12075 to 40253) | 70.31 (34.8 to 115.95) | 4723 (2865 to 7281) | 12.91 (7.82 to 19.92) | -6.12 (-6.66 to -5.57) |
| Central Europe | 4571 (2560 to 7122) | 13.39 (7.51 to 20.83) | 527 (258 to 899) | 2.53 (1.24 to 4.31) | -5.71 (-6.36 to -5.07) |
| Central Latin America | 25465 (567 to 54253) | 30.11 (0.71 to 64.12) | 4165 (324 to 9086) | 5.37 (0.31 to 11.81) | -5.1 (-5.28 to -4.92) |
| Central Sub-Saharan Africa | 88651 (1345 to 174537) | 233.72 (4.63 to 459.47) | 65732 (5884 to 133167) | 84.87 (7.65 to 171.9) | -3.64 (-4.35 to -2.93) |
| East Asia | 160476 (42836 to 294617) | 37.37 (9.9 to 68.64) | 5065 (-220 to 12171) | 1.66 (-0.08 to 3.97) | -10.17 (-10.5 to -9.83) |
| Eastern Europe | 32232 (17024 to 53932) | 50.16 (26.49 to 83.93) | 6877 (4041 to 10603) | 17.39 (10.3 to 26.63) | -3.12 (-3.58 to -2.65) |
| Eastern Sub-Saharan Africa | 258928 (33541 to 482788) | 197.51 (26.89 to 367.37) | 159925 (68583 to 273758) | 68.18 (29.26 to 116.69) | -3.7 (-4.06 to -3.34) |
| High-income Asia Pacific | 3533 (-701 to 8288) | 9.27 (-1.88 to 21.69) | 1811 (-585 to 4639) | 7.6 (-2.46 to 19.4) | -0.02 (-0.36 to 0.33) |
| High-income North America | 5299 (-845 to 17519) | 6.47 (-1.06 to 21.38) | 14896 (7114 to 25408) | 15.56 (7.36 to 26.66) | 3.3 (2.29 to 4.31) |
| North Africa and Middle East | 238935 (114060 to 389545) | 127.61 (61.03 to 208) | 93906 (55378 to 143982) | 41.46 (24.45 to 63.53) | -3.33 (-3.75 to -2.91) |
| Oceania | 5127 (760 to 9820) | 139.84 (21.25 to 267.48) | 9001 (4554 to 14010) | 127.24 (64.39 to 198.13) | 0 (-0.36 to 0.36) |
| South Asia | 2090963 (1220597 to 3117884) | 364.11 (212.58 to 543.13) | 1000619 (630708 to 1456799) | 165.88 (104.76 to 241) | -1.87 (-2.26 to -1.49) |
| Southeast Asia | 434639 (219571 to 684758) | 201.9 (101.94 to 318.09) | 159855 (90179 to 243472) | 76.1 (42.89 to 115.9) | -2.98 (-3.24 to -2.73) |
| Southern Latin America | 1655 (-1021 to 4713) | 8.73 (-5.39 to 24.86) | 398 (-121 to 1320) | 2.18 (-0.77 to 7.12) | -3.76 (-4.45 to -3.07) |
| Southern Sub-Saharan Africa | 19552 (2600 to 39454) | 71.24 (9.56 to 143.66) | 4690 (1314 to 8879) | 15.82 (4.42 to 29.96) | -4.92 (-5.37 to -4.48) |
| Tropical Latin America | 17444 (-2464 to 39743) | 27.4 (-4.07 to 62.61) | 2379 (-188 to 5749) | 3.66 (-0.35 to 8.9) | -6.62 (-6.84 to -6.41) |
| Western Europe | 4099 (-553 to 10794) | 4.79 (-0.65 to 12.46) | 6880 (344 to 16740) | 7.5 (0.16 to 18.49) | 2.64 (2.07 to 3.22) |
| Western Sub-Saharan Africa | 351012 (41511 to 674889) | 269.36 (33.15 to 516.93) | 356366 (83694 to 660267) | 121.42 (28.67 to 224.86) | -2.65 (-3.12 to -2.18) |

Abbreviations:EAPC, estimated annual percentage change, SDl, Sociodemographic Index; Ul,uncertainty interval. “ EAPC is expressed as 95% CIs.

Table.4 The YLLs cases and age-standardized YLLs rate of growth failure in children and adolescents in 1990 and 2021, along with their temporal trend.

| **Location** | Rate per 100 000(95%UI) | | 2021 | | 1990-2021 |
| --- | --- | --- | --- | --- | --- |
|  | 1990 | |  | |  |
|  | YLLs cases | The age-standardized YLLs rate | YLLs cases | The age-standardized YLLs rate | EAPC |

| **Global** | 317180998 (227994574 to 371094523) | 13894.02 (9987.32 to 16255.81) | 69147768 (35230278 to 97994188) | 2850.12 (1451.22 to 4039.78) | -4.9 (-5.21 to -4.6) |
| --- | --- | --- | --- | --- | --- |
| **SDI region** |  |  |  |  |  |
| High SDI | 581946 (407785 to 757186) | 255.74 (179.15 to 332.78) | 55206 (32673 to 81880) | 27.57 (16.23 to 41.01) | -6.03 (-6.35 to -5.71) |
| High-middle SDI | 9108332 (6716608 to 11337580) | 2660.96 (1962.07 to 3312.35) | 357952 (236209 to 486493) | 138.11 (91 to 187.85) | -9.4 (-9.63 to -9.17) |
| Middle SDI | 57934818 (42463641 to 68794282) | 7843.24 (5748.53 to 9313.52) | 5405543 (3330377 to 7368285) | 828.87 (510.03 to 1130.41) | -6.61 (-6.8 to -6.43) |
| Low-middle SDI | 124824621 (95917799 to 144395503) | 19546.37 (15019.47 to 22612.26) | 18246676 (10912173 to 24861298) | 2584.25 (1544.99 to 3521.45) | -6.06 (-6.36 to -5.75) |
| Low SDI | 124555272 (84591223 to 147933256) | 37296.1 (25333.68 to 44300.37) | 45030813 (20779406 to 64995118) | 7387.85 (3410.26 to 10662.43) | -5.08 (-5.34 to -4.81) |
| **GBD region** |  |  |  |  |  |
| Andean Latin America | 1943013 (1456453 to 2363635) | 9992.73 (7490.64 to 12155.98) | 168298 (109601 to 242547) | 741.01 (482.42 to 1068.09) | -7.73 (-7.94 to -7.53) |
| Australasia | 3443 (2183 to 5452) | 60.56 (38.38 to 95.9) | 581 (341 to 986) | 8.64 (5.07 to 14.69) | -3.91 (-4.51 to -3.3) |
| Caribbean | 1535653 (1081448 to 1902305) | 10096.26 (7110.37 to 12506.93) | 451954 (279803 to 628155) | 3170.11 (1962.3 to 4406.22) | -3.27 (-3.58 to -2.95) |
| Central Asia | 3381384 (2469799 to 4009940) | 9640.18 (7041.35 to 11432.15) | 500498 (342200 to 681603) | 1359.48 (929.52 to 1851.38) | -6.11 (-6.5 to -5.73) |
| Central Europe | 474098 (340941 to 587657) | 1409.48 (1013.58 to 1747.11) | 28293 (17394 to 39760) | 137.25 (84.29 to 192.96) | -6.76 (-7.05 to -6.46) |
| Central Latin America | 5470290 (4005894 to 6489486) | 6457.35 (4729.61 to 7659.94) | 539123 (358303 to 754467) | 722.84 (479.1 to 1013.19) | -6.27 (-6.48 to -6.06) |
| Central Sub-Saharan Africa | 13088206 (6615806 to 17423475) | 34272.64 (17333.16 to 45629.61) | 3696458 (618903 to 6783867) | 4767.9 (800.07 to 8748.72) | -6.25 (-6.87 to -5.63) |
| East Asia | 25163282 (18378614 to 30827545) | 5901.42 (4310.05 to 7229.96) | 480656 (319107 to 714809) | 162.4 (107.72 to 241.69) | -12.77 (-13.63 to -11.9) |
| Eastern Europe | 509106 (377328 to 621077) | 801.44 (593.96 to 977.73) | 36074 (24694 to 48416) | 96.43 (65.92 to 129.53) | -6.9 (-7.42 to -6.39) |
| Eastern Sub-Saharan Africa | 48917513 (33337690 to 58867691) | 36895.67 (25152.19 to 44408.03) | 13224199 (6935550 to 19389832) | 5632.92 (2955.55 to 8258.02) | -6.05 (-6.36 to -5.73) |
| High-income Asia Pacific | 60013 (40637 to 79449) | 158.7 (107.39 to 210.12) | 5210 (3121 to 7388) | 21.62 (12.86 to 30.75) | -5.08 (-5.44 to -4.72) |
| High-income North America | 64399 (39918 to 95654) | 80.61 (49.96 to 119.75) | 18202 (11480 to 27953) | 23.72 (14.84 to 36.62) | -3.01 (-3.27 to -2.74) |
| North Africa and Middle East | 17108656 (12761551 to 21652529) | 9070.49 (6765.87 to 11479.56) | 2191217 (1492439 to 2842566) | 972.38 (662.22 to 1261.45) | -6.91 (-7.19 to -6.64) |
| Oceania | 505490 (374766 to 646140) | 13671.2 (10135.44 to 17475.67) | 410666 (273552 to 557406) | 5766.31 (3841.06 to 7826.94) | -1.95 (-2.25 to -1.66) |
| South Asia | 108101575 (87551877 to 124571765) | 18697.2 (15142.41 to 21546.79) | 13359771 (9612831 to 17341697) | 2285.63 (1644.49 to 2966.88) | -6.02 (-6.37 to -5.66) |
| Southeast Asia | 26222926 (19967387 to 30963610) | 12212.93 (9299.49 to 14420.53) | 2522204 (1684510 to 3270028) | 1214.65 (810.84 to 1575.05) | -6.87 (-6.99 to -6.75) |
| Southern Latin America | 228729 (171517 to 283784) | 1206.22 (904.44 to 1496.6) | 16853 (11276 to 24177) | 105.12 (69.97 to 151.33) | -6.74 (-7.15 to -6.33) |
| Southern Sub-Saharan Africa | 3544702 (2556616 to 4206633) | 12884.07 (9293.04 to 15290.12) | 1316121 (889083 to 1755489) | 4444.78 (3002.01 to 5928.96) | -2.68 (-3.13 to -2.23) |
| Tropical Latin America | 4235741 (2796506 to 5247818) | 6729.45 (4441.83 to 8338.02) | 168416 (109405 to 235523) | 264.94 (171.89 to 370.76) | -9.55 (-9.83 to -9.27) |
| Western Europe | 57869 (34058 to 88530) | 68.33 (40.18 to 104.58) | 9808 (4878 to 16106) | 12.46 (6.16 to 20.5) | -4.2 (-4.52 to -3.87) |
| Western Sub-Saharan Africa | 56564908 (35021919 to 69697821) | 42993.07 (26621.81 to 52976.54) | 30003164 (11665905 to 44489191) | 10192.65 (3964.61 to 15112.92) | -4.49 (-4.78 to -4.19) |

Abbreviations:EAPC, estimated annual percentage change, SDl, Sociodemographic Index; Ul,uncertainty interval. “ EAPC is expressed as 95% CIs.
